# Group‐Sequential Designs With an Externally‐Driven Change of Primary Endpoint

**DOI:** 10.1002/sim.70337

**Published:** 2025-12-10

**Authors:** Amin Yarahmadi, Lori E. Dodd, Peter Horby, Thomas Jaki, Nigel Stallard

**Affiliations:** ^1^ Clinical Trials Unit Warwick Medical School University of Warwick, Coventry UK; ^2^ Clinical Trials Research and Statistics Branch National Institute of Allergy and Infectious Diseases Bethesda Maryland USA; ^3^ Pandemic Sciences Institute, Nuffield Department of Medicine University of Oxford Oxford UK; ^4^ Faculty of Informatics and Data Science University of Regensburg Regensburg Germany; ^5^ MRC Biostatistics Unit University of Cambridge Cambridge UK

**Keywords:** emerging disease clinical trial, group‐sequential stopping boundary, primary endpoint change, type I error rate spending function

## Abstract

Clinical trials conducted during the COVID‐19 pandemic demonstrated the value of adaptive design methods in emerging disease settings, when there can be considerable uncertainty around disease natural history, anticipated endpoint effect sizes and population size. In such settings, there may also be uncertainty regarding the most appropriate primary endpoint. This might lead to an externally‐driven decision to change the primary endpoint during the course of an adaptive trial. If information on the new primary endpoint is already being collected, initially as a secondary endpoint, the trial could continue with a new primary endpoint. In this case it is unclear how statistical inference on the final primary endpoint should be adjusted for interim analyses monitoring the initial primary endpoint so as to control the overall type I error rate as adjusting for monitoring as if this was based on the new endpoint could be conservative whereas failing to make any adjustment could lead to type I error rate inflation if the new and original endpoint are correlated. This paper shows how group‐sequential methods can be modified to control the type I error rate for the analysis of the new primary endpoint irrespective of the true treatment effect on the initial primary endpoint. The method is illustrated using a simulated data example based on a clinical trial of remdesivir in COVID‐19. Construction of critical values for the test of the new primary endpoint require a value for the correlation between this and the initial primary endpoint. We present simulation studies to demonstrate that the type I error rate is controlled when this value is estimated from the data on the two endpoints obtained from the trial.

## 1 Introduction

The planning and conduct of a confirmatory clinical trial generally requires advanced specification of a single primary endpoint [1]. This will be used for the primary assessment of treatment efficacy as well as for planning purposes, for example when determining the sample size. The choice of the primary endpoint is usually motivated by clinical and regulatory considerations to ensure that the trial will provide meaningful results that can lead to product registration.

If a clinical trial is being conducted in an emerging novel disease, an absence of knowledge of the natural disease course may make choice of a primary endpoint challenging. This was the case in clinical trials conducted early in the COVID‐19 pandemic, as illustrated by, for example, the ACCT‐1 trial [2]. In such a setting, a trial may start with a particular primary endpoints and, at some point during the course of the trial, scientific consensus changes so that specification of a new primary endpoint is desirable. If information on this new primary endpoint is already being collected, initially as a secondary endpoint, the trial could continue, but with a decision to change the primary endpoint in the final analysis.

As demonstrated by clinical trials conducted during the COVID‐19 pandemic, uncertainty about disease natural history, anticipated endpoint effect sizes and population size make adaptive design methods, including group‐sequential designs, particularly attractive [3, 4]. If such an approach is being used, the final analysis must be adjusted to allow for adaptations that were, or could have been, made at earlier interim analyses in order to ensure rigorous statistical inference, including control of type I error rates. If there is an externally‐driven decision to change the primary endpoint, so that interim analyses are based on the initial endpoint and the final, and possibly later interim analyses are based on the final primary endpoint, it is unclear how this adjustment should be made. The work reported below addresses this issue.

We envisage that a group‐sequential trial is designed and initiated to compare some experimental treatment with a control, with a particular primary endpoint that we will call endpoint A, with a decision made on the basis of endpoint A to continue the trial or to stop for futility or efficacy at each of a series of interim analyses. We assume that at some point during, or possibly subsequent to the end of, the trial, after at least one interim analysis has been conducted using endpoint A, a decision is made to change to some other endpoint, which we will call endpoint B. It is now desired to draw inference on the treatment effect on endpoint B, and to test the null hypothesis, $H_{0}^{\left(B\right)}:\theta^{\left(B\right)}=0$, where $\theta^{\left(B\right)}$ is a parameter summarising the effect of the treatment on endpoint B. It is assumed that this decision is driven by external factors rather than by the data observed in the trial itself. If data on endpoint B have not previously been collected, it is clearly impossible to test $H_{0}^{\left(B\right)}$, and to do so would essentially require a new trial to be initiated. Here we consider the case in which data on endpoint B is available for patients already in the trial, for example because these data have been collected as a secondary endpoint. If the trial has not previously been stopped, endpoint B will be monitored in subsequent interim analyses, with the trial stopped for efficacy or futility on the basis of this new primary endpoiint. If the trial has already stopped on the basis of efficacy or futility on endpoint A, we assume that an analysis of data on endpoint B collected up to the stopping point will be conducted.

Conducting the test(s) of $H_{0}^{\left(B\right)}$ in a conventional fashion will lead to type I error rate inflation above the nominal level if there is correlation between endpoints A and B or if the test of endpoint B is repeated. The focus of this paper is to obtain critical values for these tests so as to maintain control of the overall type I error rate.

## 2 Motivating Example

Our work is motivated by the example of the ACCT‐1 trial [2] of remdesivir in COVID‐19. The trial was initiated early in the COVID‐19 pandemic, with recruitment starting in February 2020, at a time when knowledge of the natural history of the disease was severely limited. An increase in knowledge of the disease natural history with time led to a change of primary endpoint being enacted.

The ACTT‐1 trial [2] was a double‐blind, randomised, placebo‐controlled trial study of intravenous remdesivir in adults hospitalised with COVID‐19 who exhibited signs of lower respiratory tract infection. Patients were recruited from nearly 70 trial sites located in ten different countries, with a total of 1062 patients randomised, 541 of which were assigned to receive remdesivir and 521 of which were assigned to the placebo.

Patients were treated with a loading dose of 200mg remdesivir on day 1 and then for 9 further days with daily doses of 100mg redesivir, or received placebo in a similar manner. Patients were followed up for 29 days with the initial primary endpoint set to be a patient's clinical status at day15, classified using an eight‐category ordinal scale, ranging from 1: not hospitalised and no limitations of activities to 8: death. Secondary outcomes evaluated the clinical status score at days 3, 5, 8, 11, 22, and 29. In early April 2020, following external data suggesting that the progression of COVID‐19 could be longer than initially anticipated, the primary endpoint was switched to time to recovery, censored at day 29 for patients who had died or had not recovered by that time. The initial primary outcome of the day 15 clinical status score was retained as an additional key secondary outcome.

The change of endpoint was followed by a review of the efficacy data by the data and safety monitoring board. This had originally been planned as an interim analysis but a more rapid than anticipated recruitment rate meant that recruitment had already been completed, though follow‐up was ongoing for some patients. The data and safety monitoring board recommended that results should be made available to the trial team, and these were subsequently made public. The final analysis results aligned with those from the preliminary report, indicating that remdesivir outperformed the placebo in significantly reducing recovery time for this patient group.

In the setting of a trial in an emerging disease, group‐sequental or adaptive designs are likely to be considered particularly attractive. In this paper we address the problem of how such an approach can be modified to accommodate an externally‐driven change of primary endpoint.

## 3 Construction of Group‐Sequential Stopping Boundaries Allowing for an Externally‐Driven Change in Primary Endpoint

### 3.1 Construction of the Original Group‐Sequential Boundary for Endpoint A

Suppose we initially plan a group‐sequential trial comparing some experimental treatment with a control treatment on the basis of endpoint A.

Let $\theta^{\left(A\right)}$ denote the treatment effect on endpoint A and suppose that we wish to test some null hypothesis $H_{0}^{\left(A\right)}:\theta^{\left(A\right)}=0$. Let $S_{k}^{\left(A\right)}$ and $I_{k}^{\left(A\right)}$ respecitvely denote the score statistic and observed Fisher's information for $\theta^{\left(A\right)}$ based on all data observed on endpoint A up to interim analysis k, k=1,2,… Note that the information levels, $I_{1}^{\left(A\right)},I_{2}^{\left(A\right)},\ldots$ are assumed to be independent of $S_{1}^{\left(A\right)},S_{2}^{\left(A\right)},\ldots$

We will assume that $S_{1}^{\left(A\right)},S_{2}^{\left(A\right)},\ldots$ follows a multivariate normal distribution with

(1) $$\left(\begin{array}{c} S_{1}^{\left(A\right)}\\ S_{2}^{\left(A\right)}\\ \vdots \end{array}\right)∼N\left(\left(\begin{array}{c} \theta^{\left(A\right)}I_{1}^{\left(A\right)}\\ \theta^{\left(A\right)}I_{2}^{\left(A\right)}\\ \vdots \end{array}\right),\left(\begin{array}{lll} I_{1}^{\left(A\right)} & I_{1}^{\left(A\right)} & \cdots \\ I_{1}^{\left(A\right)} & I_{2}^{\left(A\right)} & \cdots \\ \vdots & \vdots & \ddots \end{array}\right)\right).$$

This holds exactly if data are normally distributed with known variance, and has been shown to hold asymptotically in a wide range of other practical settings [5, 6].

At interim analysis k, the observed value of $S_{k}^{\left(A\right)}$ will be compared with some stopping boundary value, $u_{k}^{\left(A\right)}$. If $S_{k}^{\left(A\right)}\geq u_{k}^{\left(A\right)}$, the trial will stop and the null hypothesis $H_{0}^{\left(A\right)}$ will be rejected. Otherwise, the trial will continue, or stop without rejection of $H_{0}^{\left(A\right)}$ if $I_{k}^{\left(A\right)}\geq I_{\max}^{\left(A\right)}$ for some maximum information level, $I_{\max}^{\left(A\right)}$, set so as to give some specified power for the test of $H_{0}^{\left(A\right)}$. The trial may stop for futility at interim analysis if $S_{k}^{\left(A\right)}\leq l_{k}^{\left(A\right)}$ for some lower stopping bound value $l_{k}^{\left(A\right)}$. However, as it is often required that any futility stopping rule is non‐binding, we will ignore stopping for futility in calculation of the boundary values $u_{1}^{\left(A\right)},u_{2}^{\left(A\right)},\ldots$. This is considered in more detail in the Discussion section below.

It is desired to obtain stopping boundary critical values, $u_{1}^{\left(A\right)},u_{2}^{\left(A\right)},\ldots$, to control the overall type I error at (one‐sided) level α. As this constraint is insufficient to specify $u_{1}^{\left(A\right)},u_{2}^{\left(A\right)},\ldots$, it is common to require that the type I error rate is spent at a rate according to some specified spending function [7, 8, 9]. In detail, let ${\alpha^{*}}^{\left(A\right)}(t)$ denote an increasing function from [0,1] with ${\alpha^{*}}^{\left(A\right)}(0)=0$ and ${\alpha^{*}}^{\left(A\right)}(1)=\alpha$, specified in advance of observation of any data, then we require the type I error ‘spent’ by interim analysis k to be ${\alpha^{*}}^{\left(A\right)}(t_{k}^{\left(A\right)})$. That is

(2) $$\begin{array}{ll} & Pr_{H_{0}^{\left(A\right)}}(\text{Stop and reject}\;H_{0}^{\left(A\right)}\;\text{at or before interim analysis}\;k)\\ & \;={\alpha^{*}}^{\left(A\right)}(t_{k}^{\left(A\right)}), \end{array}$$

where $t_{k}^{\left(A\right)}=I_{k}^{\left(A\right)}/I_{\max}^{\left(A\right)}$. In particular, since ${\alpha^{*}}^{\left(A\right)}(1)=\alpha$, this ensures overall type I error rate control at the specified level.

Let $R_{k}^{\left(A\right)}$ for k=1,2,… denote the event that the trial reaches look k and stops at that look, rejecting $H_{0}^{\left(A\right)}$. Then $R_{1}^{\left(A\right)}=\{S_{1}^{\left(A\right)}\geq u_{1}^{\left(A\right)}\}$ and, for k=2,…, $R_{k}^{\left(A\right)}=C_{k}^{\left(A\right)}\cap \{S_{k}^{\left(A\right)}\geq u_{k}^{\left(A\right)}\}$ where

$$C_{k}^{\left(A\right)}{=⋂}_{r=1}^{k-1}\{S_{r}^{\left(A\right)}<u_{r}^{\left(A\right)}\}$$

is the event that the trial reaches look k (k=2,…).

The condition (2) is then that

(3) $$\overset{k}{\underset{r=1}{\sum }}Pr_{H_{0}^{\left(A\right)}}(R_{r}^{\left(A\right)})={\alpha^{*}}^{\left(A\right)}(t_{k}^{\left(A\right)}),$$

The critical values $u_{1}^{\left(A\right)},u_{2}^{\left(A\right)},\ldots$ can then be found to satisfy (3) recursively. In detail, using the multivariate normal distribution (1), we can find $u_{1}^{\left(A\right)}$ such that

$$Pr_{H_{0}^{\left(A\right)}}(R_{1}^{\left(A\right)})={\alpha^{*}}^{\left(A\right)}(t_{1}^{\left(A\right)})$$

and, for k>1, given $u_{1}^{\left(A\right)},\ldots ,u_{k-1}^{\left(A\right)}$, can find $u_{k}^{\left(A\right)}$ such that Equation (3) holds. Details are given by Jennison and Turnbull [10].

### 3.2 Construction of a Group‐Sequential Boundary With a Change of Endpoint

Suppose that part‐way through the trial designed as described above, a decision is made to change to a new primary endpoint, endpoint B. As described in the Introduction, we assume that data on endpoint B have been collected at previous interim analyses as a secondary endpoint.

Suppose that the decision to change to endpoint B is made at information time $t_{\tilde{k}}$, that is at interim analysis $\tilde{k}$, (or before interim analysis $\tilde{k}$ but after interim analysis $\tilde{k}-1$) with $1<\tilde{k}$. Thus the trial stopping rule will be based on endpoint A for looks $k=1,\ldots ,\tilde{k}-1$. If the trial has not been stopped at or before interim analysis $\tilde{k}-1$, it will continue with a stopping rule based on monitoring endpint B for looks $k=\tilde{k},\ldots$. If the trial has been stopped by interim analysis $\tilde{k}-1$, an analysis will be conducted based on the final data on endpoint B. We will consider $t_{\tilde{k}}$ such that $t_{\tilde{k}}=1$ to indicate that a decision is made to change the endpoint after the trial has concluded, with the final analysis again based on the data observed for endpoint B.

Let $\theta^{\left(B\right)}$ denote the treatment effect on endpoint B. Following the change of endpoint, we wish to test the null hypothesis $H_{0}^{\left(B\right)}:\theta^{\left(B\right)}=0$.

In order to control the overall type I error rate, we require

(4) $$Pr_{H_{0}^{\left(B\right)}}(\text{Stop and reject}\;H_{0}^{\left(B\right)})=\alpha .$$

Noting that the probability of stopping, and hence the probability of stopping and rejecting $H_{0}^{\left(B\right)}$, depends on the data on endpoint A, it is required that this holds for any $\theta_{A}$, that is that

$$\underset{\theta^{\left(A\right)}}{\sup}\left\{Pr_{H_{0}^{\left(B\right)}}(\text{Stop and reject}\;H_{0}^{\left(B\right)})\right\}=\alpha .$$

We suppose that we have a pre‐specified spending function, ${\alpha^{*}}^{\left(B\right)}$, an increasing function from [0,1] with ${\alpha^{*}}^{\left(B\right)}(0)=0$ and ${\alpha^{*}}^{\left(B\right)}(1)=\alpha$ and that it is required that

(5) $$\begin{array}{ll} & \underset{\theta^{\left(A\right)}}{\sup}\left\{Pr_{H_{0}^{\left(B\right)}}(\text{Stop and reject}\;H_{0}^{\left(B\right)}\;\text{at or before interim analysis}k)\right\}\\ & \;={\alpha^{*}}^{\left(B\right)}(t_{k}^{\left(B\right)}) \end{array}$$

where $t_{k}^{\left(B\right)}=I_{k}^{\left(B\right)}/I_{\max}^{\left(B\right)}$ with $I_{\max}^{\left(B\right)}$ the expected value of $I^{\left(B\right)}$ with a sample size for which $I^{\left(A\right)}=I_{\max}^{\left(A\right)}$.

Let $S_{k}^{\left(B\right)}$ and $I_{k}^{\left(B\right)}$ respecitvely denote the score statistic and observed Fisher's information for $\theta^{\left(B\right)}$ based on all data on endpoint B observed up to interim analysis k and assume endpoints A and B are possibly correlated such that the correlation between $S_{k}^{\left(A\right)}$ and $S_{k}^{\left(B\right)}$ is ρ. For normally distributed endpoints, this is equivalent to a correlation between the endpoints of ρ (see Friede et al. [11]).

Analogous to (1), we will assume a multivariate normal distribution for $S_{1}^{\left(A\right)},S_{2}^{\left(A\right)},\ldots ,S_{1}^{\left(B\right)},S_{2}^{\left(B\right)},\ldots$ such that

(6) $$\begin{array}{ll} & {\left(S_{1}^{\left(A\right)},S_{2}^{\left(A\right)},\ldots ,S_{1}^{\left(B\right)},S_{2}^{\left(B\right)},\ldots \right)}^{\prime }\\ & \;∼N\left({\left(\theta^{\left(A\right)}I_{1}^{\left(A\right)},\theta^{\left(A\right)}I_{2}^{\left(A\right)},\ldots ,\theta^{\left(B\right)}I_{1}^{\left(B\right)},\theta^{\left(B\right)}I_{2}^{\left(B\right)},\ldots \;\right)}^{\prime },\sum \right) \end{array}$$

with

$$\sum =\left(\begin{array}{llllll} I_{1}^{\left(A\right)} & I_{1}^{\left(A\right)} & \cdots & I_{1}^{\left(AB\right)} & I_{1}^{\left(AB\right)} & \cdots \\ I_{1}^{\left(A\right)} & I_{2}^{\left(A\right)} & \cdots & I_{1}^{\left(AB\right)} & I_{2}^{\left(AB\right)} & \cdots \\ \vdots & \vdots & \ddots & \vdots & \vdots & \ddots \\ I_{1}^{\left(AB\right)} & I_{1}^{\left(AB\right)} & \cdots & I_{1}^{\left(B\right)} & I_{1}^{\left(B\right)} & \cdots \\ I_{1}^{\left(AB\right)} & I_{2}^{\left(AB\right)} & \cdots & I_{1}^{\left(B\right)} & I_{2}^{\left(B\right)} & \cdots \\ \vdots & \vdots & \ddots & \vdots & \vdots & \ddots \end{array}\right)$$

where $I_{k}^{\left(AB\right)}=\rho {\left(I_{k}^{\left(A\right)}I_{k}^{\left(B\right)}\right)}^{1/2},k=1,2,\ldots$.

Let $u_{k}^{\left(B\right)}$ denote the critical value for the test at interim analysis k, such that $H_{0}^{\left(B\right)}$ will be rejected and the trial stopped if $S_{k}^{\left(B\right)}\geq u_{k}^{\left(B\right)}$ for $k\geq \tilde{k}$ or $H_{0}^{\left(B\right)}$ will be rejected if $S_{k}^{\left(B\right)}\geq u_{k}^{\left(B\right)}$ if the trial stopped at look k because $S_{k}^{\left(A\right)}\geq u_{k}^{\left(A\right)}$ for $k<\tilde{k}$.

Let $R_{k}^{\left(B\right)}$ denote the event that the trial reaches look k and stops at that look, rejecting $H_{0}^{\left(B\right)}$. Recalling that this can occur either for $k\geq \tilde{k}$, with stopping and rejection of $H_{0}^{\left(B\right)}$ based on monitoring of endpoint B, or for $k<\tilde{k}$, when the trial has already stopped with rejection of $H_{0}^{\left(A\right)}$, we have

(7) $$R_{k}^{\left(B\right)}=\left\{\begin{array}{ll} R_{k}^{\left(A\right)}\cap \{S_{k}^{\left(B\right)}\geq u_{k}^{\left(B\right)}\}\; & k<\tilde{k}\\ C_{k}^{\left(A\right)}\cap \{S_{k}^{\left(B\right)}\geq u_{k}^{\left(B\right)}\}\; & k=\tilde{k}\\ C_{\tilde{k}}^{\left(A\right)}\cap C_{\tilde{k},k}^{\left(B\right)}\cap \{S_{k}^{\left(B\right)}\geq u_{k}^{\left(B\right)}\}\; & k>\tilde{k} \end{array}\right.$$

with, for $k>\tilde{k}$,

$$C_{\tilde{k},k}^{\left(B\right)}{=⋂}_{r=\tilde{k}}^{k-1}\{S_{r}^{\left(B\right)}<u_{r}^{\left(B\right)}\}.$$

Condition (5) can thus be rewritten as

(8) $$\underset{\theta^{\left(A\right)}}{\sup}\left\{\overset{k}{\underset{r=1}{\sum }}Pr_{H_{0}^{\left(B\right)}}(R_{r}^{\left(B\right)})\right\}={\alpha^{*}}^{\left(B\right)}(t_{k}^{\left(B\right)}).$$

Critical values $u_{1}^{\left(B\right)},\ldots ,u_{K}^{\left(B\right)}$ can again be found recursively so as to satisfy (8) with $u_{1}^{\left(B\right)}$ such that

(9) $$\underset{\theta_{A}}{\sup}\left\{Pr_{H_{0}^{\left(B\right)}}(R_{1}^{\left(B\right)})\right\}={\alpha^{*}}^{\left(B\right)}(t_{1}^{\left(B\right)})$$

and, for k>1, the value of $u_{k}^{\left(B\right)}$ to satisfy (8) found given $u_{1}^{\left(B\right)},\ldots ,u_{k-1}^{\left(B\right)}$.

For k=1, the supremum in (9) corresponds to $\theta_{A}\to \infty$ so that

$$Pr_{\theta^{\left(A\right)}}(R_{1}^{\left(A\right)})\to 1$$

and

$$\underset{\theta_{A}}{\sup}\;Pr_{H_{0}^{\left(B\right)}}(R_{1}^{\left(B\right)})=Pr_{H_{0}^{\left(B\right)}}(S_{1}^{\left(B\right)}\geq u_{1}^{\left(B\right)}).$$

For $k=\tilde{k}=2$, it possible to find the value of $\theta_{A}$ at which the supremum in (8) occurs analytically (see Supporting Information). Otherwise the supremum must be found numerically, for example using the one‐dimensional optimisation routine optimize in R [12].

Calculation of the critical values $u_{1}^{\left(B\right)},u_{2}^{\left(B\right)},\ldots$ require the variance‐covariance matrix ∑ in Equation (6) to be known. The information levels may be $I_{k}^{\left(A\right)}$ and $I_{k}^{\left(B\right)}$ are estimated from the data available at the kth interim analysis using expressions given by Whitehead [13]. The correlation, ρ, between the test statistics $S_{k}^{\left(A\right)}$ and $S_{k}^{\left(B\right)}$ will also generally be unknown. An estimated value might be available based on historical data, or alternatively ρ can be estimated from the data available from the trial. As noted above, for normally distributed endpoints, ρ is equal to the correlation between the endpoints A and B in each treatment group [11]. In this case a direct estimate of ρ can be obtained. For other endpoints types, such as binary or time‐to‐event data, it can be estimated using a bootstrap procedure, resampling pairs of endpoints with replacement from each the control and treatment groups and calculating $S^{\left(A\right)}$ and $S^{\left(B\right)}$ values, with their correlation estimated from a large number of resampled replicates. Since boundary values $u_{k}^{\left(B\right)}$ for $k\leq \tilde{k}$ are only required at the point when the trial stops, the construction of these boundary values can be based on a value of ρ estimated using all data from the look at which the trial stops if this is prior to look $\tilde{k}$, or from the data at look $\tilde{k}$ if the trial has not stopped at this point. This is illustrated in detail in the example give in Section 4.1. The simulation study reported in Section 4.2 examines the type I error rate when ρ is estimated from the data for normally distributed data.

## 4 Numerical Example and Simulation Study

### 4.1 Detailed Numerical Example

In order to illustrate the method proposed above, we simulated trial data using the model proposed by Dodd et al. [14]. This enabled simulation of data on an the ordinal eight‐level scale as used in the study described in Section 2 above [2] over a 28 day follow‐up period for patients with and without treatment. Reflecting a situation similar to that described in Section 2 above, we assumed that the initial endpoint was the binary outcome corresponding to attainment of a score of 2 or less after 15 days, where a score of 2 = ambulatory with limitation on activities, and that subsequently a decision was made to change the endpoint to be time to recovery (score 1), with this censored after 28 days. Expressions for $S^{\left(A\right)},I^{\left(A\right)},S^{\left(B\right)}$ and $I^{\left(B\right)}$ for binary and time‐to‐event endpoints are given by Whitehead [13].

We suppose that the initial design was constructed to have five stages with equal numbers of patients, with these randomised 1:1 to treatment and placebo control groups, with an α‐spending function of the form ${\alpha^{*}}^{\left(A\right)}(t)=\alpha t$ (see Hwang et al. [9]) with one‐sided type I error rate α=0.025, and that is desired to have power of 0.9 to detect an increase in the proportion of patients attaining a score of 2 or less within 15 days from 0.5 in the control group to 0.58 in the treatment group. This corresponds to $I_{\max}^{\left(A\right)}=114.6$. From Whitehead [13], this requires a total maximum sample size of 1844 patients, with 184 patients per group per stage for stages 1, 3 and 5 and 185 patients per group per stage for stages 2 and 4. The full simulated data set is given in the Supporting Information.

We will illustrate the proposed method using two example data sets. In the first, we imagine that the trial proceeds based on endpoint A, but that subsequently a decision is made to change the primary endpoint to be endpoint B. Suppose that the data on endpoint A for the first three stages of the trial are as shown in Table 1. The corresponding values of $S_{k}^{\left(A\right)}$ and $I_{k}^{\left(A\right)}$ for k=1,…,3 are shown, together with the critical values for $S^{\left(A\right)}$ calculated using the method described in Section 3.1. At the third interim analysis, after observation of 553 patients per group we have $S_{3}^{\left(A\right)}>u_{3}^{\left(A\right)}$ and the trial stops. The null hypothesis $H_{0}^{\left(A\right)}$ would have been rejected at this point.

**TABLE 1:** Summary of results and critical boundary values for looks 1 to 3 for endpoint A in the simulated example data set.

	Total successes					
k	Control	Treated	$I_{k}^{\left(A\right)}$	$u_{k}^{\left(A\right)}$	$u_{k}^{\left(A\right)}/\surd I_{k}^{\left(A\right)}$	$S_{k}^{\left(A\right)}$
1	93	110	22.75	12.3	2.58	8.5
2	192	221	45.47	16.8	2.50	14.5
3	281	331	68.34	19.9	2.41	25.0

Following the decision to change to endpoint B. Test statitics $S_{3}^{\left(B\right)}$ and $I_{3}^{\left(B\right)}$ can be calculated based on the observed time to recovery data from the 1106 patients recruited to the trial. Assuming a recovery rate of approximately 40%, with a total maximum sample size of 1844 patients, the maximum information on endpoint B, $I_{\max}^{\left(B\right)}$, was calculated to be 184.5.

**FIGURE 1:**
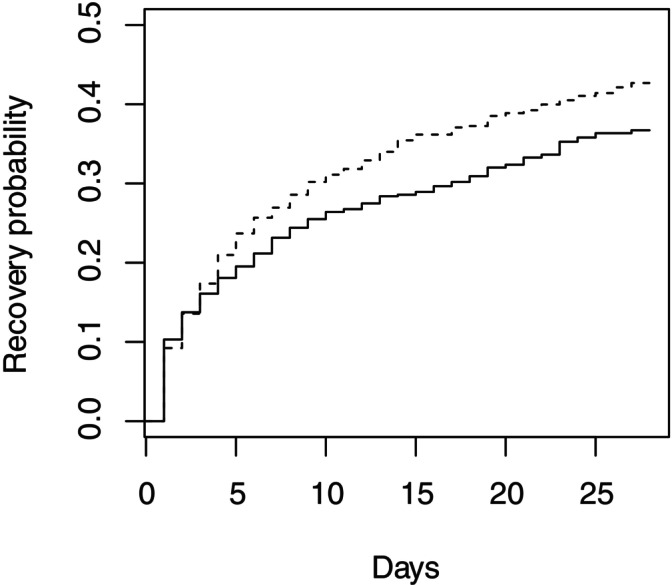
Kaplan–Meier curves illustrating simulated example time to recovery data in control (solid line) and treated (dashed line) groups at interim analysis 3 (when trial stopped with k=5).

The method above illustrates how to construct the correct critical value, $u_{3}^{\left(B\right)}$, with $S_{3}^{\left(B\right)}$ should be compared. Beacuse the trial could have stopped at an earlier interim, calculation of $u_{3}^{\left(B\right)}$ requires the values of $I_{1}^{\left(B\right)}$ and $I_{2}^{\left(B\right)}$. For this simulated data set, these are given in Table 2 along with the cumulative number of recovery events in each arm observed at each interim analysis. Calculation of the value of $u_{3}^{\left(B\right)}$, and the values of $u_{1}^{\left(B\right)}$ and $u_{2}^{\left(B\right)}$, on which it depends, require a value for ρ, the correlation between $S_{k}^{\left(A\right)}$ and $S_{k}^{\left(B\right)}$. This was estimated using a bootstrap approach as described above. In this case an estimated correlation of 0.715 was obtained. Resulting values for $u_{1}^{\left(B\right)},\ldots ,u_{3}^{\left(B\right)}$ are shown in Table 2, together with standardised boundary values given by $u_{i}^{\left(B\right)}/\surd I_{i}^{\left(B\right)},i=1,\ldots ,3$ and the values of $\theta^{\left(A\right)}$ for which the supremum in (8) occurs, denoted $\sup(\theta^{\left(A\right)})$. The simulated times to recovery for the 1106 patients observed up to interim analysis 3 are illustrated in the Kaplan‐Meier plot shown in Figure 1. The value of $S_{3}^{\left(B\right)}$ is 23.13. As this exceeds $u_{3}^{\left(B\right)}$, the null hypothesis $H_{0}^{\left(B\right)}$ can be rejected.

**TABLE 2:** Summary of results and critical boundary values for endpoint B in the simulated example data set with $\tilde{k}=5$.

	Total recoveries				Boundary with $\tilde{k}=5$			
k	Control	Treated	$I_{k}^{\left(B\right)}$	$S_{k}^{\left(B\right)}$	$\hat{\rho }$	$\sup(\theta^{\left(A\right)})$	$u_{k}^{\left(B\right)}$	$u_{k}^{\left(B\right)}/\surd I_{k}^{\left(B\right)}$
1	71	76	35.35	1.60	0.715^a^	∞	15.4	2.59
2	140	154	70.53	7.71	0.715^a^	0.2396	18.9	2.26
3	203	241	106.49	23.13	0.715	0.1838	21.6	2.09

^a^ Estimate from later look used as boundary value calculated retrospectively.

**FIGURE 2:**
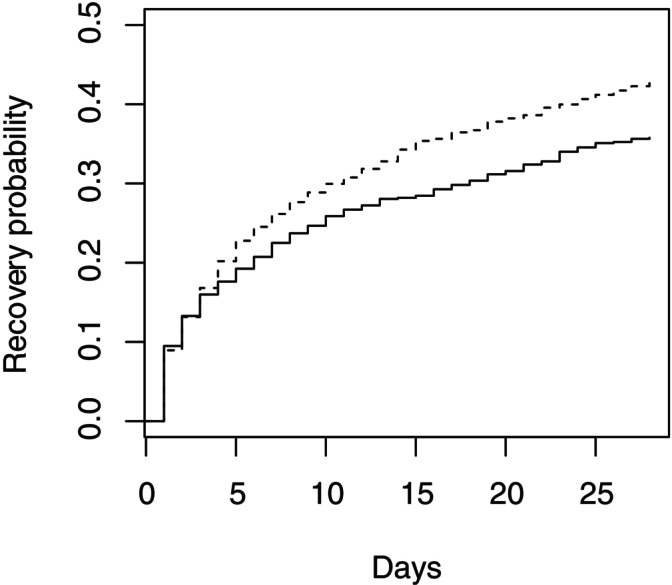
Kaplan–Meier curves illustrating simulated example time to recovery data in control (solid line) and treated (dashed line) groups at interim analysis 4 (when trial stopped with $\tilde{k}=2$).

In the second example data set we envisage data as above but suppose that a decision was taken to change to endpoint B after the first interim analysis, that is $\tilde{k}=2$. Since the trial has not stopped at this point, the trial continues, monitoring endpoint B from interim analysis 2 onwards. At each interim analysis, the value of $I_{k}^{\left(B\right)}$ is calculated and used to calculate $u_{k}^{\left(B\right)}$ as described above, based on an estimated value of ρ. The values of $I_{k}^{\left(B\right)}$, estimated values of ρ, values of $\theta^{\left(A\right)}$ corresponding to the supremum in (9), and resulting values of $u_{k}^{\left(B\right)}$ are given in Table 3. In this case, the critical values $u_{k}^{\left(B\right)}$ are larger than those when $\tilde{k}=5$ as they are adjusted for the possibility of earlier stopping when this is more often based on observation of a large value of endpoint B than of a large value of endpoint A. In this case the value of $S_{3}^{\left(B\right)}$ of 23.13 does not exceed the critical value $u_{3}^{\left(B\right)}$, so the trial would continue to interim analysis 4. The simulated times to recovery for the 1484 patients observed up to interim analysis 4 are illustrated in the Kaplan‐Meier plot shown in Figure 2. The value of $S_{4}^{\left(B\right)}$ is 32.35. As this exceeds $u_{4}^{\left(B\right)}$, the trial would stop and $H_{0}^{\left(B\right)}$ would be rejected.

**TABLE 3:** Summary of results and critical boundary values for endpoint B in the simulated example data set with $\tilde{k}=2$.

	Total recoveries				Boundary with $\tilde{k}=2$			
k	Control	Treated	$I_{k}^{\left(B\right)}$	$S_{k}^{\left(B\right)}$	$\hat{\rho }$	$\sup(\theta^{\left(A\right)})$	$u_{k}^{\left(B\right)}$	$u_{k}^{\left(B\right)}/\surd I_{k}^{\left(B\right)}$
1	71	76	35.35	1.60	0.721^a^	∞	15.4	2.59
2	140	154	70.53	7.71	0.721	0.0769	19.9	2.37
3	203	241	106.49	23.13	0.705	0.0030	24.5	2.37
4	264	318	139.91	32.25	0.729	−1.5836	27.7	2.34

^a^ Estimate from later look used as boundary value calculated retrospectively.

### 4.2 Simulation Study

A simulation study was conducted to assess the properties of the method proposed above and to compare it with possible alternative approaches.

A group‐sequential trial was initially designed for a normally distributed endpoint, endpoint A, with up to five stages planned with these equally spaced in terms of information. The parameter of interest, $\theta^{\left(A\right)}$, was defined to be the standardised difference in means between the treated and control group. Expressions for $S^{\left(A\right)}$ and $I^{\left(A\right)}$ are given by Whitehead [13].

The trial was designed with an alpha‐spending function ${\alpha^{*}}^{\left(A\right)}(t^{\left(A\right)})=\alpha t^{\left(A\right)}$ for an overall one‐sided type one error rate α=0.025, and power 0.9 to detect a treatment effect of size $\theta^{\left(A\right)}=0.5$. This requires $I_{\max}^{\left(A\right)}=47.75$, equivalent to a total sample size per treatment group of 95.5. This was rounded up to 96, with 19 patients included per treatment arm in each of stages 1 to 4 and 20 patients per treatment arm included in stage 5.

For looks $k=1,\ldots ,\tilde{k}$, for $\tilde{k}$ equal to 3 and 5, the observed values of $S_{k}^{\left(A\right)}$ are compared with critical values for the group sequential test, with the trial stopped if $S_{k}^{\left(A\right)}\geq u_{k}^{\left(A\right)}$.

It was assumed that at look $\tilde{k}$ a decision was made to change to a new endpoint, endpoint B, with this also normally distributed. If the trial had already stopped, the final data for endpoint B would be analysed. Otherwise, the trial would continue with the same sample size per treatment group per stage with endpoint B monitored, so that the test has nominal power of 0.9 for a standardised treatment difference on endpoint B, $\theta^{\left(B\right)}$, also equal to 0.5. Critical values $u^{\left(B\right)}$ will be obtained to satisfy Equation (9) with ${\alpha^{*}}^{\left(B\right)}(t^{\left(B\right)})=\alpha t^{\left(B\right)}$ with the correlation between the test statisctics, ρ, which for normally distributed endpoints is just the correlation between the endpoints, estimated from the observed data. Note that for $\tilde{k}=5$, since this is the last planned interim analysis, the stopping time is determined entirely by endpoint A with endpoint B analysed for the resulting final sample.

In order to assess error rate control for a range of values of $\theta^{\left(A\right)}$, simulations were conducted with $\theta^{\left(A\right)}$ ranging from −0.3 to 0.5, with $\theta^{\left(B\right)}$ equal to 0, that is under the null hypothesis, 0.3 and 0.5, that is, with true effect of treatment B smaller than or of equal size to the effect on treatment A for which the trial was originally powered, with a true correlation between endpoints A and B, ρ of 0.7 or 0.3. As $\theta^{\left(A\right)}$ and $\theta^{\left(B\right)}$ are defined as standardised treatment differences, the variances for the normal endpoints A and B can be set arbitrarily, so were set to 1 in both cases in the simulation study.

Tables 4 and 5 give simulation results from 10 000 simulations under each of the scenarios described above for ρ equal to 0.7 and 0.3 respectively. Estimated error rates for the approach described above, labelled ‘Corrected test’ are compared with those for two other alternative approaches to testing $H_{0}^{\left(B\right)}$. The first, labelled ‘Naive test’ is to compare $S_{k}^{\left(B\right)}$ with a standard critical value of $1.96\surd I_{k}$ at the look at which the trial stops if this is less than $\tilde{k}$, or at every interim analysis after $\tilde{k}$ if the trial has not stopped, in the latter case stopping as soon as a critical value is exceeded. The second alternative approach, labelled ‘Group‐sequential’ is to compare $S_{k}^{\left(B\right)}$ to a group‐sequential boundary obtained using $I^{\left(B\right)}$ and spending function ${\alpha^{*}}^{\left(B\right)}$ ignoring the fact that the trial might have been stopped early because of observation of a large value of endpoint A.

**TABLE 4:** Simulation results for ρ=0.7.

		Corrected test			Naive test			Group‐sequential test		
		Type I	Power	Power	Type I	Power	Power	Type I	Power	Power
$\tilde{k}$	$\theta^{\left(A\right)}$	error	$\theta^{\left(B\right)}=0.3$	$\theta^{\left(B\right)}=0.5$	error	$\theta^{\left(B\right)}=0.3$	$\theta^{\left(B\right)}=0.5$	error	$\theta^{\left(B\right)}=0.3$	$\theta^{\left(B\right)}=0.5$
2	−0.3	0.0225	0.4834	0.9001	0.0549	0.6226	0.9475	0.0201	0.4720	0.8954
	−0.1	0.0227	0.4836	0.8999	0.0552	0.6227	0.9476	0.0203	0.4716	0.8954
	0	0.0224	0.4820	0.8992	0.0556	0.6225	0.9475	0.0200	0.4706	0.8949
	0.1	0.0221	0.4800	0.8974	0.0567	0.6216	0.9472	0.0197	0.4690	0.8930
	0.3	0.0198	0.4633	0.8819	0.0575	0.6116	0.9426	0.0180	0.4524	0.8775
	0.5	0.0152	0.4083	0.8288	0.0524	0.5762	0.9216	0.0138	0.3990	0.8248
3	−0.3	0.0262	0.4921	0.9045	0.0483	0.6051	0.9442	0.0196	0.4595	0.8927
	−0.1	0.0263	0.4919	0.9045	0.0485	0.6053	0.9443	0.0196	0.4591	0.8928
	0	0.0259	0.4909	0.9040	0.0493	0.6054	0.9442	0.0192	0.4580	0.8923
	0.1	0.0254	0.4879	0.9015	0.0513	0.6036	0.9437	0.0192	0.4534	0.8895
	0.3	0.0220	0.4503	0.8803	0.0514	0.5794	0.9359	0.0161	0.4119	0.8609
	0.5	0.0134	0.3359	0.7806	0.0415	0.4885	0.8845	0.0097	0.2926	0.7389
4	−0.3	0.0242	0.5016	0.9109	0.0376	0.5806	0.9400	0.0153	0.4444	0.8882
	−0.1	0.0246	0.5011	0.9109	0.0385	0.5809	0.9401	0.0155	0.4441	0.8883
	0	0.0246	0.4999	0.9105	0.0402	0.5812	0.9400	0.0153	0.4428	0.8878
	0.1	0.0249	0.4964	0.9080	0.0430	0.5797	0.9395	0.0161	0.4367	0.8850
	0.3	0.0217	0.4499	0.8847	0.0436	0.5488	0.9296	0.0133	0.3767	0.8503
	0.5	0.0125	0.2941	0.7598	0.0364	0.4183	0.8574	0.0081	0.2173	0.6785
5	−0.3	0.0205	0.4943	0.9154	0.0261	0.5411	0.9317	0.0117	0.4143	0.8788
	−0.1	0.0216	0.4939	0.9153	0.0280	0.5414	0.9318	0.0124	0.4141	0.8789
	0	0.0229	0.4927	0.9147	0.0312	0.5416	0.9317	0.0129	0.4130	0.8785
	0.1	0.0256	0.4903	0.9122	0.0366	0.5414	0.9312	0.0143	0.4079	0.8758
	0.3	0.0229	0.4519	0.8884	0.0417	0.5158	0.9213	0.0119	0.3473	0.8394
	0.5	0.0123	0.2879	0.7582	0.0359	0.3906	0.8452	0.0077	0.1895	0.6534

**TABLE 5:** Simulation results for ρ=0.3.

		Corrected test			Naive test			Group‐sequential test		
		Type I	Power	Power	Type I	Power	Power	Type I	Power	Power
$\tilde{k}$	$\theta^{\left(A\right)}$	error	$\theta^{\left(B\right)}=0.3$	$\theta^{\left(B\right)}=0.5$	error	$\theta^{\left(B\right)}=0.3$	$\theta^{\left(B\right)}=0.5$	error	$\theta^{\left(B\right)}=0.3$	$\theta^{\left(B\right)}=0.5$
2	−0.3	0.0252	0.4759	0.9003	0.0596	0.6218	0.9473	0.0217	0.4640	0.8962
	−0.1	0.0252	0.4747	0.8993	0.0596	0.6210	0.9468	0.0217	0.4631	0.8953
	0	0.0252	0.4733	0.8972	0.0596	0.6195	0.9456	0.0217	0.4617	0.8931
	0.1	0.0249	0.4698	0.8925	0.0594	0.6168	0.9425	0.0214	0.4581	0.8885
	0.3	0.0240	0.4499	0.8654	0.0593	0.5985	0.9252	0.0208	0.4393	0.8615
	0.5	0.0211	0.4052	0.8026	0.0560	0.5584	0.8823	0.0183	0.3953	0.7991
3	−0.3	0.0257	0.4836	0.9051	0.0484	0.6037	0.9437	0.0184	0.4528	0.8937
	−0.1	0.0255	0.4823	0.9044	0.0484	0.6025	0.9432	0.0182	0.4511	0.8925
	0	0.0255	0.4806	0.9011	0.0485	0.5999	0.9411	0.0182	0.4486	0.8893
	0.1	0.0258	0.4737	0.8949	0.0486	0.5939	0.9365	0.0181	0.4408	0.8816
	0.3	0.0244	0.4325	0.8465	0.0474	0.5520	0.9010	0.0169	0.3942	0.8218
	0.5	0.0203	0.3446	0.7292	0.0425	0.4596	0.8086	0.0127	0.2967	0.6818
4	−0.3	0.0252	0.5074	0.9164	0.0376	0.5797	0.9394	0.0145	0.4370	0.8909
	−0.1	0.0250	0.5056	0.9157	0.0376	0.5783	0.9389	0.0142	0.4350	0.8896
	0	0.0254	0.5030	0.9122	0.0380	0.5755	0.9366	0.0143	0.4323	0.8859
	0.1	0.0254	0.4941	0.9050	0.0378	0.5673	0.9312	0.0143	0.4207	0.8752
	0.3	0.0261	0.4416	0.8469	0.0372	0.5129	0.8855	0.0125	0.3548	0.7975
	0.5	0.0209	0.3281	0.7045	0.0349	0.3967	0.7663	0.0091	0.2353	0.6128
5	−0.3	0.0231	0.5107	0.9228	0.0262	0.5380	0.9304	0.0109	0.4092	0.8822
	−0.1	0.0231	0.5095	0.9222	0.0265	0.5368	0.9300	0.0109	0.4075	0.8809
	0	0.0240	0.5064	0.9187	0.0270	0.5339	0.9277	0.0113	0.4044	0.8771
	0.1	0.0250	0.4972	0.9109	0.0286	0.5256	0.9216	0.0117	0.3924	0.8656
	0.3	0.0273	0.4488	0.8507	0.0319	0.4729	0.8731	0.0104	0.3238	0.7835
	0.5	0.0224	0.3326	0.7048	0.0329	0.3690	0.7530	0.0079	0.2071	0.5886

The results in Table 4 show that using the naive testing approach leads to inflation of the one‐sided type I error rate above the nominal 0.025 level, particularly for smaller $\tilde{k}$. This is as expected, as in this case, there is no adjustment for the repeated significance testing of endpoint B at looks $k\geq \tilde{k}$. The error rate inflation for the naive testing approach when $\tilde{k}=5$ arises because of the correlation between endpoints A and B. The fact that the test stops when $S_{k}^{\left(A\right)}\geq u_{k}^{\left(A\right)}$ means that this is likely to arise for an unusually large value of $S^{\left(A\right)}$, and hence, due to the correlation, for an unusually large value of $S_{k}^{\left(B\right)}$, leading to the type I error rate inflation observed. As the true value of $\theta^{\left(A\right)}$ becomes large stopping at the first interim analysis becomes increasing likely so that the type I error rate inflation for the naive test of $H_{0}^{\left(B\right)}$ is reduced; it would be expected that this would approach the nominal 0.025 level as $\theta^{\left(A\right)}\to \infty$, irrespective of the value of $\tilde{k}$. For $\tilde{k}=5$, as the true value of $\theta^{\left(A\right)}$ becomes very small, continuing to the last interim analysis becomes increasing likely so that the type I error rate inflation for the naive test of $H_{0}^{\left(B\right)}$ is again reduced and would be expected to approach 0.025 as $\theta^{\left(A\right)}\to -\infty$.

The group‐sequential test of $H_{0}^{\left(B\right)}$ with critical values constructed ignoring the monitoring of endpoint A prior to look $\tilde{k}$, is conservative, as the boundary over‐corrects for the sequential testing. This is particularly true for larger $\tilde{k}$ values.

The sequential testing boundaries constructed as above control the type I error rate at or below the nominal level as intended for all values of $\theta^{\left(A\right)}$. As the test is constructed to control the type I error rate for any $\theta_{A}$, there is some conservatism, particuarly when the trie value of $\theta^{\left(A\right)}$ is large. The power to detect an effect of size $\theta^{\left(B\right)}=0.5$ is also reduced below the nominal level of 0.9 for larger values of $\theta^{\left(A\right)}$, with this most marked for larger $\tilde{k}$, as the trial then tends to stop with a smaller sample size than if endpoint B has beeen used from the outset. Unsurprisingly, the power is reduced for smaller $\theta^{\left(B\right)}$ for all values of $\theta^{\left(A\right)}$ and $\tilde{k}$.

The results in Table 5 are similar to those in Table 4, showing that the true value of ρ has relatively little impact on the properties of the testing procedures, though the smaller value of ρ leads to the method proposed above being slightly less conservative when $\theta^{\left(A\right)}$ is large and to a slightly lower degree of type I error rate inflation for the naive test when $\theta^{\left(A\right)}$ is close to 0.

Additional simulation results giving the probability that the trial stops at each interim analysis are given in the Supporting Information.

## 5 Discussion

Clinical trials conducted during the COVID‐19 pandemic demonstrated the value of adaptive design methods in settings in which there is uncertainty about disease natural history, anticipated endpoint effect sizes and population size [3, 4]. In such a setting, there may also be uncertainty regarding the most appropriate endpoint to use to assess the impact of treatments under investigation. As it would be undesirable to delay the start of clinical trials, this might lead to an externally‐driven decision to change the primary endpoint during the course of a trial [2]. This is a relatively unusual scenario, but as the motivating example described above illustrates, can arise in trials in emerging novel pathogens.

If no data have been collected on the new primary endpoint, endpoint B, in the ongoing trial then in order to be able to draw inference on the treatment effect on endpoint B will require a new trial. If, however, data on endpoint B were collected as a secondary endpoint, this data could be analysed at the end of the trial to draw inference on endpoint B. As demonstrated by the simulation study reported above, the use of inappropriate statistical methods in such a setting could lead to either inflated type I error rates for a test of $H_{0}^{\left(B\right)}$, the null hypothesis that there is no effect on endpoint B, or alternatively to uneccessary conservatism and a resulting lack of power. It is therefore important to develop statistical approaches specific to this setting. This was the aim of this paper.

The method proposed above calculates adjusted critical values for a test of $H_{0}^{\left(B\right)}$ allowing for the fact that in its earlier stages the trial was monitored, and may have been stopped, using endpoint A. Construction of boundary values requires specification of the correlation between the test statistics based on the two endpoints, ρ. In the simulations reported above, this was estimated based on the data observed in the trial. The simulation results suggest that this does not lead to type I error rate inflation, though this is not guaranteed for smaller sample sizes when the correlation is less accurately estimated. If this was a particular concern, since assuming a larger correlation leads to a more conservative test, a larger value such as, for example, an upper 95% confidence limit, could deliberatly be used.

The approach described above adjusts only for stopping for a positive effect (that is stopping for efficacy) on endpoint A in the early stages or endpoint B in the later stages of the trial. A lower (futility) stopping rule could be added [15, 16]. If this was to be considered binding, the method described could be modified by defining the events $C_{k}^{\left(A\right)}$ and $C_{\tilde{k},k}^{\left(B\right)}$ to also require that the test statistic exceeded the lower boundary at looks 1,…,k−1. If a futility stopping rule for endpoint B is introduced but not allowed for in the construction of $u_{1}^{\left(B\right)},u_{2}^{\left(B\right)},\ldots$, this will reduce the probability of rejecting $H_{0}^{\left(B\right)}$ and hence lead to a conservative boundary, and associated loss of power. Introduction of a futility stopping rule for endpoint A will also lead to conservatism in the test of $H_{0}^{\left(B\right)}$ provided the correlation, ρ, is non‐negative. It would generally be reasonable to assume that this is the case, but if such an assumption was considered unreasonable, use of a non‐binding futility stopping rule for endpoint A should be avoided to prevent type I error rate inflation.

The critical values $u_{1}^{\left(B\right)},u_{2}^{\left(B\right)},\ldots$ depend on the specified rate at which the type I error rate is spent, given by ${\alpha^{*}}^{\left(B\right)}(t)$. In the examples above we have used ${\alpha^{*}}^{\left(B\right)}(t)={\alpha^{*}}^{\left(A\right)}(t)$ so that the rate of error spending is equal to that originally planned for endpoint A. Whilst in priniciple a different alpha‐spending function could be used, type I error rate control requires that this is specified independently of any observed trial data. As a change of endpoint would not typically be envisaged in advance, it is hard to imagine how a form for ${\alpha^{*}}^{\left(B\right)}(t)$ that differs from ${\alpha^{*}}^{\left(A\right)}(t)$ could be justified. In particular, specifying ${\alpha^{*}}^{\left(B\right)}(t)$ at the point at which the trial stops or the endpoint change occurs in such a way as to allocate less alpha to earlier interim analyses could result in overall type I error rate inflation for the test of $H_{0}^{\left(B\right)}$; for the setting in which $\tilde{k}$ corresponds to the final analysis, this is equivalent to the ‘naive test’ considered in the simulation study above. Changes in the timing of future analyses based on the estimate of $\theta_{(B})$ could also lead to error rate inflation (see Proschan et al. [17]), though the sample size for the trial following the change of endpoint could be altered based on information from outside the trial, for example to give power for a smaller treatment effect.

An alternative approach to that described above could be based on the conditional error principal [18]. Using this approach, boundary values for interim analyses after a change in endpoint can be found based on the conditional type I error given the data on endpoint A at the time when the endpoint is changed. The conditioning on the endpoint A data from interim analyses with $k<\tilde{k}$ means that the decision to make a change of endpoint can depend on these data rather than being considered to be driven by external factors as in the work above. This will lead to more stringent test boundary values.

The work presented above has focussed on control of the type I error rate for the test of $H_{0}^{\left(B\right)}$. In addition, estimation of $\theta^{\left(B\right)}$ presents an important inferential problem, with conventional point estimates likely to be biased and conventional confidence intervals likely to have inaccurate coverage. A number of methods have been proposed for point and interval estimation following group sequential trials [19, 20, 21] upon which further research work in this area could be based.

The method proposed in this paper assumes that the change in endpoint is externally‐driven, that is that the decision to change the endpoint is not influenced by any data observed in the trial. It is also assumed that the initial endpoint, endpoint A, is no longer of interest as a primary endpoint. The focus is thus on control of the type I error rate for the test of $H_{0}^{\left(B\right)}$ for any value of $\theta^{\left(A\right)}$, the unknown treatment effect on endpoint A. This is in contrast to methods that allow co‐primary endpoints, for which control of the familywise error rate for the family $\left\{H_{0}^{\left(A\right)},H_{0}^{\left(B\right)}\right\}$ might be of more concern [22].

Although the work described has been motivated, and illustrated, by clinical trials in COVID‐19, this is not the only area of application. Scientific consensus on suitable endpoints has also changed in other diseases, with recent examples including other severe infections [23], dengue [24] and pulmonary arterial hypertension [25]. These changes in endpoint would lead to similar challenges problems to those described above for any ongoing group‐sequential trials. A specific example of the primary endpoint being changed during the course of an ongoing trial is the IMASOY trial in bubonic plague [26].

## Funding

This work was supported by the UK Medical Research Council (MRC) under grant numbers: MR/V038419/1, MC_UU_00002/14, and MC_UU_00040/03.

## Conflicts of Interest

The authors declare no conflicts of interest.

## Supporting information




**Data S1.** Supporting Information.

## Data Availability

Simulated data used in Section 4.1 are available in the Supporting Information of this article.
