# Supplementary material for: Group‐Sequential Designs With an Externally‐Driven Change of Primary Endpoint
Source: Stat Med. 2025 Dec 10;44(28-30):e70337. doi: 10.1002/sim.70337 (PMC12694703; doi:10.1002/sim.70337)
Supplement: Supplementary file 1 — Data S1. Supporting Information. [file SIM-44-0-s001.pdf]

Group-sequential designs with an externally-driven change of primary endpoint

Amin Yarahmadi, Lori E. Dodd, Peter Horby, Thomas Jaki, Nigel Stallard*

Supplementary material

*Address for correspondence:
Nigel Stallard, Clinical Trials Unit, Warwick Medical School,
University of Warwick, Coventry, CV4 7AL, UK.
(N.Stallard@warwick.ac.uk)

An analytic expression for the value of $\theta^{(A)}$ to maximise the error rate for testing $H_0^{(B)}$ when $k = \tilde{k} = 2$

For $k = \tilde{k} = 2$, the condition (8) in the main paper becomes

$$\sup_{\theta^{(A)}} \left\{ \sum_{r=1}^{2} Pr_{H_0^{(B)}}(\mathscr{R}_r^{(B)}) \right\} = \alpha^{*(B)}(t_2^{(B)}).$$

In order to obtain $u_2^{(B)}$, it is therefore necessary to find the value of $\theta^{(A)}$ for which this supremum occurs, that is for which

$$\tilde{\alpha} = Pr_{\theta^{(A)},\theta^{(B)}=0}\left(S_1^{(A)} \geq u_1^{(A)}, S_1^{(B)} \geq u_1^{(B)}\right) + Pr_{\theta^{(A)},\theta^{(B)}=0}\left(S_1^{(A)} < u_1^{(A)}, S_2^{(B)} \geq u_2^{(B)}\right),$$

or equivalently,

$$\tilde{\alpha} = \int_{u_1^{(B)}}^{\infty} Pr_{\theta^{(A)},\theta^{(B)}=0}\left(S_1^{(A)} \geq u_1^{(A)} \mid S_1^{(B)} = s\right) f_{S_1^{(B)}}(s)ds + \int_{u_2^{(B)}}^{\infty} Pr_{\theta^{(A)},\theta^{(B)}=0}\left(S_1^{(A)} < u_1^{(A)} \mid S_2^{(B)} = s\right) f_{S_2^{(B)}}(s)ds$$

is maximised.

Differentiating $\tilde{\alpha}$ with respect to $\theta^{(A)}$ and exchanging the order of integration and differentiation, gives

$$\begin{aligned} \frac{d\tilde{\alpha}}{d\theta^{(A)}} = &\int_{u_1^{(B)}}^{\infty} \frac{d}{d\theta^{(A)}} \left\{ Pr_{\theta^{(A)},\theta^{(B)}=0}\left(S_1^{(A)} \geq u_1^{(A)} \mid S_1^{(B)} = s\right) \right\} f_{S_1^{(B)}}(s)ds \\ &+ \int_{u_2^{(B)}}^{\infty} \frac{d}{d\theta^{(A)}} \left\{ Pr_{\theta^{(A)},\theta^{(B)}=0}\left(S_1^{(A)} < u_1^{(A)} \mid S_2^{(B)} = s\right) \right\} f_{S_2^{(B)}}(s)ds. \end{aligned} \tag{A.1}$$

From (6) in the main paper we have, for $\theta^{(B)} = 0$,

$$\begin{pmatrix} S_1^{(A)} \\ S_1^{(B)} \\ S_2^{(B)} \end{pmatrix} \sim N\left(\begin{pmatrix} \theta_A I_1^{(A)} \\ 0 \\ 0 \end{pmatrix}, \begin{pmatrix} I_1^{(A)} & \rho\sqrt{I_1^{(A)} I_1^{(B)}} & \rho_2\sqrt{I_1^{(A)} I_2^{(B)}} \\ \rho\sqrt{I_1^{(A)} I_1^{(B)}} & I_1^{(B)} & I_1^{(B)} \\ \rho_2\sqrt{I_1^{(A)} I_2^{(B)}} & I_1^{(B)} & I_2^{(B)} \end{pmatrix} \right)$$

where $\rho_2 = \rho {I_1^{(B)}}^{1/2} {I_2^{(B)}}^{-1/2}$ is the correlation between $S_1^{(A)}$ and $S_2^{(B)}$. Thus

$$\begin{aligned} S_1^{(A)} \mid S_1^{(B)} = s &\sim N\left(\theta^{(A)} I_1^{(A)} + \rho\sqrt{I_1^{(A)}/I_1^{(B)}}s, (1-\rho^2)I_1^{(A)}\right) \\ S_1^{(A)} \mid S_2^{(B)} = s &\sim N\left(\theta^{(A)} I_1^{(A)} + \rho_2\sqrt{I_1^{(A)}/I_2^{(B)}}s, (1-\rho_2^2)I_1^{(A)}\right). \end{aligned}$$

Denoting by ϕ and Φ the standard normal density and distribution functions respectively,the derivative

(A.1) is thus given by

$$\frac{d\tilde{\alpha}}{d\theta^{(A)}} = \int_{u_1^{(B)}}^{\infty} \frac{d}{d\theta^{(A)}}\left[1-\Phi\left\{\frac{u_1^{(A)}-\left(\theta^{(A)}I_1^{(A)}+\rho\sqrt{\frac{I_1^{(A)}}{I_1^{(B)}}}s\right)}{\sqrt{(1-\rho^2)I_1^{(A)}}}\right\}\right]\frac{1}{\sqrt{I_1^{(B)}}}\phi\left(\frac{s}{\sqrt{I_1^{(B)}}}\right)ds$$

$$+\int_{u_2^{(B)}}^{\infty} \frac{d}{d\theta^{(A)}}\left[\Phi\left\{\frac{u_1^{(A)}-\left(\theta^{(A)}I_1^{(A)}+\rho_2\sqrt{\frac{I_1^{(A)}}{I_2^{(B)}}}s\right)}{\sqrt{(1-\rho_2^2)I_1^{(A)}}}\right\}\right]\frac{1}{\sqrt{I_2^{(B)}}}\phi\left(\frac{s}{\sqrt{I_2^{(B)}}}\right)$$

$$=\int_{u_1^{(B)}}^{\infty} \frac{I_1^{(A)}}{\sqrt{(1-\rho^2)I_1^{(A)}}}\phi\left\{\frac{u_1^{(A)}-\left(\theta^{(A)}I_1^{(A)}+\rho\sqrt{\frac{I_1^{(A)}}{I_1^{(B)}}}s\right)}{\sqrt{(1-\rho^2)I_1^{(A)}}}\right\}\frac{1}{\sqrt{I_1^{(B)}}}\phi\left(\frac{s}{\sqrt{I_1^{(B)}}}\right)ds$$

$$+\int_{u_2^{(B)}}^{\infty} \frac{-I_1^{(A)}}{\sqrt{(1-\rho_2^2)I_1^{(A)}}}\phi\left\{\frac{u_1^{(A)}-\left(\theta^{(A)}I_1^{(A)}+\rho_2\sqrt{\frac{I_1^{(A)}}{I_2^{(B)}}}s\right)}{\sqrt{(1-\rho_2^2)I_1^{(A)}}}\right\}\frac{1}{\sqrt{I_2^{(B)}}}\phi\left(\frac{s}{\sqrt{I_2^{(B)}}}\right)ds$$

The result given in Section 1 of Bromiley[1] enables this to be rewritten as

$$\frac{d\tilde{\alpha}}{d\theta^{(A)}} =\sqrt{I_1^{(A)}}\phi\left\{\frac{u_1^{(A)}-\theta^{(A)}I_1^{(A)}}{\sqrt{I_1^{(A)}}}\right\}\int_{u_1^{(B)}}^{\infty}\frac{1}{\sqrt{(1-\rho^2)I_1^{(B)}}}\phi\left\{\frac{s-\frac{\rho\left(u_1^{(A)}-\theta^{(A)}I_1^{(A)}\right)\sqrt{I_1^{(B)}}}{\sqrt{I_1^{(A)}}}}{\sqrt{(1-\rho^2)I_1^{(B)}}}\right\}ds$$

$$-\sqrt{I_1^{(A)}}\phi\left\{\frac{u_1^{(A)}-\theta^{(A)}I_1^{(A)}}{\sqrt{I_1^{(A)}}}\right\}\int_{u_2^{(B)}}^{\infty}\frac{1}{\sqrt{(1-\rho_2^2)I_2^{(B)}}}\phi\left\{\frac{s-\frac{\rho_2\left(u_1^{(A)}-\theta^{(A)}I_1^{(A)}\right)\sqrt{I_2^{(B)}}}{\sqrt{I_1^{(A)}}}}{\sqrt{(1-\rho_2^2)I_2^{(B)}}}\right\}ds$$

$$=\sqrt{I_1^{(A)}}\phi\left\{\frac{u_1^{(A)}-\theta^{(A)}I_1^{(A)}}{\sqrt{I_1^{(A)}}}\right\}\left[\Phi\left\{\frac{u_2^{(B)}-\frac{\rho_2\left(u_1^{(A)}-\theta^{(A)}I_1^{(A)}\right)\sqrt{I_2^{(B)}}}{\sqrt{I_1^{(A)}}}}{\sqrt{(1-\rho_2^2)I_2^{(B)}}}\right\}-\Phi\left\{\frac{u_1^{(B)}-\frac{\rho\left(u_1^{(A)}-\theta^{(A)}I_1^{(A)}\right)\sqrt{I_1^{(B)}}}{\sqrt{I_1^{(A)}}}}{\sqrt{(1-\rho^2)I_1^{(B)}}}\right\}\right].$$

Hence $d\tilde{\alpha}/d\theta^{(A)} = 0$ when

$$\Phi\left\{\frac{u_2^{(B)}-\frac{\rho_2\left(u_1^{(A)}-\theta^{(A)}I_1^{(A)}\right)\sqrt{I_2^{(B)}}}{\sqrt{I_1^{(A)}}}}{\sqrt{(1-\rho_2^2)I_2^{(B)}}}\right\}=\Phi\left\{\frac{u_1^{(B)}-\frac{\rho\left(u_1^{(A)}-\theta^{(A)}I_1^{(A)}\right)\sqrt{I_1^{(B)}}}{\sqrt{I_1^{(A)}}}}{\sqrt{(1-\rho^2)I_1^{(B)}}}\right\}$$

that is when

$$\frac{u_2^{(B)}-\frac{\rho_2\left(u_1^{(A)}-\theta^{(A)}I_1^{(A)}\right)\sqrt{I_2^{(B)}}}{\sqrt{I_1^{(A)}}}}{\sqrt{(1-\rho_2^2)I_2^{(B)}}}=\frac{u_1^{(B)}-\frac{\rho\left(u_1^{(A)}-\theta^{(A)}I_1^{(A)}\right)\sqrt{I_1^{(B)}}}{\sqrt{I_1^{(A)}}}}{\sqrt{(1-\rho^2)I_1^{(B)}}}$$

or equivalently, when

$$\theta^{(A)}=\frac{\left(\frac{\frac{u_2^{(B)}}{\sqrt{I_2^{(B)}}}-\rho_2\frac{u_1^{(A)}}{\sqrt{I_1^{(A)}}}}{\sqrt{1-\rho_2^2}}-\frac{\frac{u_1^{(B)}}{\sqrt{I_1^{(B)}}}-\rho\frac{u_1^{(A)}}{\sqrt{I_1^{(A)}}}}{\sqrt{1-\rho^2}}\right)}{\sqrt{I_1^{(A)}}\left(\frac{\rho}{\sqrt{1-\rho^2}}-\frac{\rho_2}{\sqrt{1-\rho_2^2}}\right)}. \qquad (A.2)$$

References

1. Bromiley, P.A. (2014) Products and convolutions of Gaussian probability density functions. Tina Memo No. 2003-003. Imaging Science and Biomedical Engineering Divison, Medical School, University of Manchester. `http://www.lucamartino.altervista.org/2003-003.pdf`. Accessed 13 March 2025.

Additional simulation results

The following tables give the probability of stopping and the probability of stopping and rejecting $H_0^{(B)}$ at each look for the simulated trials summarised in Tables 4 and 5 in the main paper. These are given for the proposed method, the naive test of $H_0^{(B)}$and the group-sequential test of $H_0^{(B)}$ designed ignoring the change of endpoint, both under the null ($\theta^{(B)} = 0$) and alternative ($\theta^{(B)} = 0.5$) hypotheses, for $\tilde{k} = 2, 3, 4$ and 5. In each case, the sum over the five looks of the probabilities of stopping and rejecting $H_0^{(B)}$ gives the type I error or power reported in Tables 4 and 5 of the main paper.

Table 1: Simulation results for $\tilde{k} = 2$ with $\rho = 0.7$

		$\theta^{(B)} = 0$						$\theta^{(B)} = 0.5$					
		Corrected test		Naive test		Group-sequential test		Corrected test		Naive test		Group-sequential test	
$\theta^{(A)}$	Look	Pr(stop)	Pr(reject)	Pr(stop)	Pr(reject)	Pr(stop)	Pr(reject)	Pr(stop)	Pr(reject)	Pr(stop)	Pr(reject)	Pr(stop)	Pr(reject)
-0.3	1	0.0006	0.0001	0.0006	0.0004	0.0006	0.0001	0.0006	0.0004	0.0006	0.0006	0.0006	0.0004
	2	0.0098	0.0098	0.0273	0.0273	0.0067	0.0067	0.4268	0.4268	0.5845	0.5845	0.3668	0.3668
	3	0.0060	0.0060	0.0137	0.0137	0.0060	0.0060	0.2159	0.2159	0.2028	0.2028	0.2490	0.2490
	4	0.0041	0.0041	0.0096	0.0096	0.0041	0.0041	0.1593	0.1593	0.1056	0.1056	0.1752	0.1752
	5	0.0071	0.0071	0.0083	0.0083	0.0070	0.0070	0.0977	0.0977	0.0540	0.0540	0.1040	0.1040
-0.1	1	0.0029	0.0009	0.0029	0.0018	0.0029	0.0009	0.0029	0.0026	0.0029	0.0029	0.0029	0.0026
	2	0.0094	0.0094	0.0264	0.0264	0.0064	0.0064	0.4246	0.4246	0.5823	0.5823	0.3646	0.3646
	3	0.0059	0.0059	0.0134	0.0134	0.0058	0.0058	0.2158	0.2158	0.2028	0.2028	0.2490	0.2490
	4	0.0038	0.0038	0.0096	0.0096	0.0040	0.0040	0.1590	0.1590	0.1056	0.1056	0.1752	0.1752
	5	0.0071	0.0071	0.0083	0.0083	0.0070	0.0070	0.0979	0.0979	0.0540	0.0540	0.1040	0.1040
0	1	0.0075	0.0015	0.0075	0.0041	0.0075	0.0015	0.0075	0.0067	0.0075	0.0074	0.0075	0.0067
	2	0.0089	0.0089	0.0254	0.0254	0.0063	0.0063	0.4201	0.4201	0.5777	0.5777	0.3605	0.3605
	3	0.0056	0.0056	0.0129	0.0129	0.0055	0.0055	0.2154	0.2154	0.2028	0.2028	0.2487	0.2487
	4	0.0039	0.0039	0.0096	0.0096	0.0040	0.0040	0.1595	0.1595	0.1056	0.1056	0.1752	0.1752
	5	0.0071	0.0071	0.0082	0.0082	0.0068	0.0068	0.0975	0.0975	0.0540	0.0540	0.1038	0.1038
0.1	1	0.0155	0.0024	0.0155	0.0073	0.0155	0.0024	0.0155	0.0127	0.0155	0.0150	0.0155	0.0127
	2	0.0080	0.0080	0.0236	0.0236	0.0057	0.0057	0.4133	0.4133	0.5702	0.5702	0.3543	0.3543
	3	0.0056	0.0056	0.0126	0.0126	0.0055	0.0055	0.2147	0.2147	0.2025	0.2025	0.2475	0.2475
	4	0.0035	0.0035	0.0091	0.0091	0.0037	0.0037	0.1595	0.1595	0.1055	0.1055	0.1748	0.1748
	5	0.0068	0.0068	0.0081	0.0081	0.0065	0.0065	0.0972	0.0972	0.0540	0.0540	0.1037	0.1037
0.3	1	0.0565	0.0039	0.0565	0.0151	0.0565	0.0039	0.0565	0.0367	0.0565	0.0507	0.0565	0.0367
	2	0.0059	0.0059	0.0189	0.0189	0.0044	0.0044	0.3804	0.3804	0.5335	0.5335	0.3233	0.3233
	3	0.0048	0.0048	0.0108	0.0108	0.0045	0.0045	0.2110	0.2110	0.2001	0.2001	0.2422	0.2422
	4	0.0032	0.0032	0.0086	0.0086	0.0032	0.0032	0.1573	0.1573	0.1049	0.1049	0.1729	0.1729
	5	0.0065	0.0065	0.0077	0.0077	0.0063	0.0063	0.0965	0.0965	0.0534	0.0534	0.1024	0.1024
0.5	1	0.1526	0.0051	0.1526	0.0222	0.1526	0.0051	0.1526	0.0769	0.1526	0.1247	0.1526	0.0769
	2	0.0030	0.0030	0.0117	0.0117	0.0020	0.0020	0.3136	0.3136	0.4532	0.4532	0.2626	0.2626
	3	0.0030	0.0030	0.0079	0.0079	0.0027	0.0027	0.1965	0.1965	0.1907	0.1907	0.2232	0.2232
	4	0.0025	0.0025	0.0070	0.0070	0.0024	0.0024	0.1491	0.1491	0.1008	0.1008	0.1632	0.1632
	5	0.0056	0.0056	0.0062	0.0062	0.0054	0.0054	0.0927	0.0927	0.0522	0.0522	0.0989	0.0989

Table 2: Simulation results for $\tilde{k} = 3$ with $\rho = 0.7$

		$\theta^{(B)} = 0$						$\theta^{(B)} = 0.5$					
		Corrected test		Naive test		Group-sequential test		Corrected test		Naive test		Group-sequential test	
$\theta^{(A)}$	Look	Pr(stop)	Pr(reject)	Pr(stop)	Pr(reject)	Pr(stop)	Pr(reject)	Pr(stop)	Pr(reject)	Pr(stop)	Pr(reject)	Pr(stop)	Pr(reject)
-0.3	1	0.0006	0.0001	0.0006	0.0004	0.0006	0.0001	0.0006	0.0004	0.0006	0.0006	0.0006	0.0004
	2	0.0000	0.0000	0.0000	0.0000	0.0000	0.0000	0.0000	0.0000	0.0000	0.0000	0.0000	0.0000
	3	0.0144	0.0144	0.0271	0.0271	0.0086	0.0086	0.6651	0.6651	0.7561	0.7561	0.5894	0.5894
	4	0.0058	0.0058	0.0121	0.0121	0.0046	0.0046	0.1502	0.1502	0.1275	0.1275	0.1931	0.1931
	5	0.0059	0.0059	0.0087	0.0087	0.0063	0.0063	0.0888	0.0888	0.0600	0.0600	0.1098	0.1098
-0.1	1	0.0029	0.0009	0.0029	0.0018	0.0029	0.0009	0.0029	0.0026	0.0029	0.0029	0.0029	0.0026
	2	0.0012	0.0004	0.0012	0.0005	0.0012	0.0002	0.0012	0.0012	0.0012	0.0012	0.0012	0.0012
	3	0.0137	0.0137	0.0259	0.0259	0.0081	0.0081	0.6615	0.6615	0.7527	0.7527	0.5861	0.5861
	4	0.0054	0.0054	0.0116	0.0116	0.0042	0.0042	0.1504	0.1504	0.1275	0.1275	0.1931	0.1931
	5	0.0059	0.0059	0.0087	0.0087	0.0062	0.0062	0.0888	0.0888	0.0600	0.0600	0.1098	0.1098
0	1	0.0075	0.0015	0.0075	0.0041	0.0075	0.0015	0.0075	0.0067	0.0075	0.0074	0.0075	0.0067
	2	0.0049	0.0017	0.0049	0.0022	0.0049	0.0011	0.0049	0.0049	0.0049	0.0049	0.0049	0.0049
	3	0.0119	0.0119	0.0231	0.0231	0.0068	0.0068	0.6533	0.6533	0.7446	0.7446	0.5782	0.5782
	4	0.0051	0.0051	0.0113	0.0113	0.0039	0.0039	0.1506	0.1506	0.1275	0.1275	0.1929	0.1929
	5	0.0057	0.0057	0.0086	0.0086	0.0059	0.0059	0.0885	0.0885	0.0598	0.0598	0.1096	0.1096
0.1	1	0.0155	0.0024	0.0155	0.0073	0.0155	0.0024	0.0155	0.0127	0.0155	0.0150	0.0155	0.0127
	2	0.0160	0.0024	0.0160	0.0044	0.0160	0.0016	0.0160	0.0154	0.0160	0.0158	0.0160	0.0151
	3	0.0106	0.0106	0.0209	0.0209	0.0062	0.0062	0.6350	0.6350	0.7259	0.7259	0.5601	0.5601
	4	0.0046	0.0046	0.0104	0.0104	0.0035	0.0035	0.1500	0.1500	0.1272	0.1272	0.1921	0.1921
	5	0.0054	0.0054	0.0083	0.0083	0.0055	0.0055	0.0884	0.0884	0.0598	0.0598	0.1095	0.1095
0.3	1	0.0565	0.0039	0.0565	0.0151	0.0565	0.0039	0.0565	0.0367	0.0565	0.0507	0.0565	0.0367
	2	0.0896	0.0061	0.0896	0.0120	0.0896	0.0034	0.0896	0.0822	0.0896	0.0858	0.0896	0.0745
	3	0.0050	0.0050	0.0108	0.0108	0.0026	0.0026	0.5305	0.5305	0.6173	0.6173	0.4592	0.4592
	4	0.0032	0.0032	0.0075	0.0075	0.0024	0.0024	0.1455	0.1455	0.1236	0.1236	0.1848	0.1848
	5	0.0038	0.0038	0.0060	0.0060	0.0038	0.0038	0.0854	0.0854	0.0585	0.0585	0.1057	0.1057
0.5	1	0.1526	0.0051	0.1526	0.0222	0.1526	0.0051	0.1526	0.0769	0.1526	0.1247	0.1526	0.0769
	2	0.2448	0.0048	0.2448	0.0109	0.2448	0.0019	0.2448	0.1854	0.2448	0.2079	0.2448	0.1539
	3	0.0011	0.0011	0.0026	0.0026	0.0006	0.0006	0.3240	0.3240	0.3936	0.3936	0.2695	0.2695
	4	0.0008	0.0008	0.0029	0.0029	0.0006	0.0006	0.1201	0.1201	0.1052	0.1052	0.1475	0.1475
	5	0.0016	0.0016	0.0029	0.0029	0.0015	0.0015	0.0742	0.0742	0.0531	0.0531	0.0911	0.0911

Table 3: Simulation results for $\tilde{k} = 4$ with $\rho = 0.7$

		$\theta^{(B)} = 0$						$\theta^{(B)} = 0.5$					
		Corrected test		Naive test		Group-sequential test		Corrected test		Naive test		Group-sequential test	
$\theta^{(A)}$	Look	Pr(stop)	Pr(reject)	Pr(stop)	Pr(reject)	Pr(stop)	Pr(reject)	Pr(stop)	Pr(reject)	Pr(stop)	Pr(reject)	Pr(stop)	Pr(reject)
-0.3	1	0.0006	0.0001	0.0006	0.0004	0.0006	0.0001	0.0006	0.0004	0.0006	0.0006	0.0006	0.0004
	2	0.0000	0.0000	0.0000	0.0000	0.0000	0.0000	0.0000	0.0000	0.0000	0.0000	0.0000	0.0000
	3	0.0001	0.0000	0.0001	0.0000	0.0001	0.0000	0.0001	0.0001	0.0001	0.0001	0.0001	0.0001
	4	0.0167	0.0167	0.0268	0.0268	0.0085	0.0085	0.8219	0.8219	0.8685	0.8685	0.7642	0.7642
	5	0.0074	0.0074	0.0104	0.0104	0.0067	0.0067	0.0885	0.0885	0.0708	0.0708	0.1235	0.1235
-0.1	1	0.0029	0.0009	0.0029	0.0018	0.0029	0.0009	0.0029	0.0026	0.0029	0.0029	0.0029	0.0026
	2	0.0012	0.0004	0.0012	0.0005	0.0012	0.0002	0.0012	0.0012	0.0012	0.0012	0.0012	0.0012
	3	0.0012	0.0007	0.0012	0.0009	0.0012	0.0004	0.0012	0.0012	0.0012	0.0012	0.0012	0.0012
	4	0.0152	0.0152	0.0249	0.0249	0.0075	0.0075	0.8174	0.8174	0.8641	0.8641	0.7598	0.7598
	5	0.0074	0.0074	0.0104	0.0104	0.0065	0.0065	0.0885	0.0885	0.0707	0.0707	0.1235	0.1235
0	1	0.0075	0.0015	0.0075	0.0041	0.0075	0.0015	0.0075	0.0067	0.0075	0.0074	0.0075	0.0067
	2	0.0049	0.0017	0.0049	0.0022	0.0049	0.0011	0.0049	0.0049	0.0049	0.0049	0.0049	0.0049
	3	0.0051	0.0020	0.0051	0.0024	0.0051	0.0009	0.0051	0.0051	0.0051	0.0051	0.0051	0.0051
	4	0.0123	0.0123	0.0213	0.0213	0.0059	0.0059	0.8055	0.8055	0.8521	0.8521	0.7478	0.7478
	5	0.0071	0.0071	0.0102	0.0102	0.0059	0.0059	0.0883	0.0883	0.0705	0.0705	0.1233	0.1233
0.1	1	0.0155	0.0024	0.0155	0.0073	0.0155	0.0024	0.0155	0.0127	0.0155	0.015	0.0155	0.0127
	2	0.0160	0.0024	0.0160	0.0044	0.0160	0.0016	0.0160	0.0154	0.0160	0.0158	0.0160	0.0151
	3	0.0190	0.0047	0.0190	0.0057	0.0190	0.0027	0.0190	0.0190	0.0190	0.0190	0.0190	0.0189
	4	0.0095	0.0095	0.0164	0.0164	0.0043	0.0043	0.7726	0.7726	0.8193	0.8193	0.7154	0.7154
	5	0.0059	0.0059	0.0092	0.0092	0.0051	0.0051	0.0883	0.0883	0.0704	0.0704	0.1229	0.1229
0.3	1	0.0565	0.0039	0.0565	0.0151	0.0565	0.0039	0.0565	0.0367	0.0565	0.0507	0.0565	0.0367
	2	0.0896	0.0061	0.0896	0.0120	0.0896	0.0034	0.0896	0.0822	0.0896	0.0858	0.0896	0.0745
	3	0.1081	0.0064	0.1081	0.0078	0.1081	0.0021	0.1081	0.1043	0.1081	0.1050	0.1081	0.0994
	4	0.0028	0.0028	0.0050	0.0050	0.0015	0.0015	0.5771	0.5771	0.6201	0.6201	0.5252	0.5252
	5	0.0025	0.0025	0.0037	0.0037	0.0024	0.0024	0.0844	0.0844	0.0680	0.0680	0.1145	0.1145
0.5	1	0.1526	0.0051	0.1526	0.0222	0.1526	0.0051	0.1526	0.0769	0.1526	0.1247	0.1526	0.0769
	2	0.2448	0.0048	0.2448	0.0109	0.2448	0.0019	0.2448	0.1854	0.2448	0.2079	0.2448	0.1539
	3	0.2350	0.0020	0.2350	0.0024	0.2350	0.0006	0.2350	0.1975	0.2350	0.2057	0.2350	0.1639
	4	0.0003	0.0003	0.0004	0.0004	0.0001	0.0001	0.2380	0.2380	0.2663	0.2663	0.2035	0.2035
	5	0.0003	0.0003	0.0005	0.0005	0.0004	0.0004	0.0620	0.0620	0.0528	0.0528	0.0803	0.0803

Table 4: Simulation results for $\tilde{k} = 5$ with $\rho = 0.7$

		$\theta^{(B)} = 0$						$\theta^{(B)} = 0.5$					
		Corrected test		Naive test		Group-sequential test		Corrected test		Naive test		Group-sequential test	
$\theta^{(A)}$	Look	Pr(stop)	Pr(reject)	Pr(stop)	Pr(reject)	Pr(stop)	Pr(reject)	Pr(stop)	Pr(reject)	Pr(stop)	Pr(reject)	Pr(stop)	Pr(reject)
-0.3	1	0.0006	0.0001	0.0006	0.0004	0.0006	0.0001	0.0006	0.0004	0.0006	0.0006	0.0006	0.0004
	2	0.0000	0.0000	0.0000	0.0000	0.0000	0.0000	0.0000	0.0000	0.0000	0.0000	0.0000	0.0000
	3	0.0001	0.0000	0.0001	0.0000	0.0001	0.0000	0.0001	0.0001	0.0001	0.0001	0.0001	0.0001
	4	0.0001	0.0001	0.0001	0.0001	0.0001	0.0001	0.0001	0.0001	0.0001	0.0001	0.0001	0.0001
	5	0.0203	0.0203	0.0256	0.0256	0.0115	0.0115	0.9148	0.9148	0.9309	0.9309	0.8782	0.8782
-0.1	1	0.0029	0.0009	0.0029	0.0018	0.0029	0.0009	0.0029	0.0026	0.0029	0.0029	0.0029	0.0026
	2	0.0012	0.0004	0.0012	0.0005	0.0012	0.0002	0.0012	0.0012	0.0012	0.0012	0.0012	0.0012
	3	0.0012	0.0007	0.0012	0.0009	0.0012	0.0004	0.0012	0.0012	0.0012	0.0012	0.0012	0.0012
	4	0.0007	0.0005	0.0007	0.0005	0.0007	0.0005	0.0007	0.0007	0.0007	0.0007	0.0007	0.0007
	5	0.0191	0.0191	0.0243	0.0243	0.0104	0.0104	0.9096	0.9096	0.9258	0.9258	0.8732	0.8732
0	1	0.0075	0.0015	0.0075	0.0041	0.0075	0.0015	0.0075	0.0067	0.0075	0.0074	0.0075	0.0067
	2	0.0049	0.0017	0.0049	0.0022	0.0049	0.0011	0.0049	0.0049	0.0049	0.0049	0.0049	0.0049
	3	0.0051	0.0020	0.0051	0.0024	0.0051	0.0009	0.0051	0.0051	0.0051	0.0051	0.0051	0.0051
	4	0.0053	0.0022	0.0053	0.0022	0.0053	0.0012	0.0053	0.0053	0.0053	0.0053	0.0053	0.0053
	5	0.0155	0.0155	0.0203	0.0203	0.0082	0.0082	0.8927	0.8927	0.9090	0.9090	0.8565	0.8565
0.1	1	0.0155	0.0024	0.0155	0.0073	0.0155	0.0024	0.0155	0.0127	0.0155	0.015	0.0155	0.0127
	2	0.016	0.0024	0.0160	0.0044	0.0160	0.0016	0.0160	0.0154	0.0160	0.0158	0.0160	0.0151
	3	0.0190	0.0047	0.0190	0.0057	0.0190	0.0027	0.0190	0.0190	0.0190	0.0190	0.0190	0.0189
	4	0.0202	0.0045	0.0202	0.0039	0.0202	0.0018	0.0202	0.0202	0.0202	0.0202	0.0202	0.0202
	5	0.0116	0.0116	0.0153	0.0153	0.0058	0.0058	0.8449	0.8449	0.8612	0.8612	0.8089	0.8089
0.3	1	0.0565	0.0039	0.0565	0.0151	0.0565	0.0039	0.0565	0.0367	0.0565	0.0507	0.0565	0.0367
	2	0.0896	0.0061	0.0896	0.0120	0.0896	0.0034	0.0896	0.0822	0.0896	0.0858	0.0896	0.0745
	3	0.1081	0.0064	0.1081	0.0078	0.1081	0.0021	0.1081	0.1043	0.1081	0.1050	0.1081	0.0994
	4	0.1147	0.0044	0.1147	0.0043	0.1147	0.0013	0.1147	0.1137	0.1147	0.1135	0.1147	0.1102
	5	0.0021	0.0021	0.0025	0.0025	0.0012	0.0012	0.5515	0.5515	0.5663	0.5663	0.5186	0.5186
0.5	1	0.1526	0.0051	0.1526	0.0222	0.1526	0.0051	0.1526	0.0769	0.1526	0.1247	0.1526	0.0769
	2	0.2448	0.0048	0.2448	0.0109	0.2448	0.0019	0.2448	0.1854	0.2448	0.2079	0.2448	0.1539
	3	0.2350	0.0020	0.2350	0.0024	0.2350	0.0006	0.2350	0.1975	0.2350	0.2057	0.2350	0.1639
	4	0.1596	0.0004	0.1596	0.0004	0.1596	0.0001	0.1596	0.1447	0.1596	0.1444	0.1596	0.1234
	5	0.0000	0.0000	0.0000	0.0000	0.0000	0.0000	0.1537	0.1537	0.1625	0.1625	0.1353	0.1353

Table 5: Simulation results for $\tilde{k} = 2$ with $\rho = 0.3$

		$\theta^{(B)} = 0$						$\theta^{(B)} = 0.5$					
		Corrected test		Naive test		Group-sequential test		Corrected test		Naive test		Group-sequential test	
$\theta^{(A)}$	Look	Pr(stop)	Pr(reject)	Pr(stop)	Pr(reject)	Pr(stop)	Pr(reject)	Pr(stop)	Pr(reject)	Pr(stop)	Pr(reject)	Pr(stop)	Pr(reject)
-0.3	1	0.0005	0.0000	0.0005	0.0000	0.0005	0.0000	0.0005	0.0002	0.0005	0.0003	0.0005	0.0002
	2	0.0102	0.0102	0.0274	0.0274	0.0065	0.0065	0.4333	0.4333	0.5879	0.5879	0.3697	0.3697
	3	0.0052	0.0052	0.0139	0.0139	0.0057	0.0057	0.2046	0.2046	0.2001	0.2001	0.2432	0.2432
	4	0.0035	0.0035	0.0100	0.0100	0.0035	0.0035	0.1646	0.1646	0.1055	0.1055	0.1804	0.1804
	5	0.0063	0.0063	0.0083	0.0083	0.0060	0.0060	0.0976	0.0976	0.0535	0.0535	0.1027	0.1027
-0.1	1	0.0026	0.0001	0.0026	0.0004	0.0026	0.0001	0.0026	0.0012	0.0026	0.0018	0.0026	0.0012
	2	0.0102	0.0102	0.0272	0.0272	0.0065	0.0065	0.4315	0.4315	0.5861	0.5861	0.3681	0.3681
	3	0.0051	0.0051	0.0138	0.0138	0.0056	0.0056	0.2044	0.2044	0.1999	0.1999	0.2429	0.2429
	4	0.0035	0.0035	0.0099	0.0099	0.0035	0.0035	0.1646	0.1646	0.1055	0.1055	0.1804	0.1804
	5	0.0063	0.0063	0.0083	0.0083	0.0060	0.0060	0.0976	0.0976	0.0535	0.0535	0.1027	0.1027
0	1	0.0063	0.0002	0.0063	0.0007	0.0063	0.0002	0.0063	0.0023	0.0063	0.0041	0.0063	0.0023
	2	0.0102	0.0102	0.0271	0.0271	0.0065	0.0065	0.4292	0.4292	0.5834	0.5834	0.3664	0.3664
	3	0.0051	0.0051	0.0136	0.0136	0.0056	0.0056	0.2039	0.2039	0.1995	0.1995	0.2419	0.2419
	4	0.0034	0.0034	0.0099	0.0099	0.0034	0.0034	0.1642	0.1642	0.1051	0.1051	0.1799	0.1799
	5	0.0063	0.0063	0.0083	0.0083	0.0060	0.0060	0.0976	0.0976	0.0535	0.0535	0.1026	0.1026
0.1	1	0.0152	0.0005	0.0152	0.0019	0.0152	0.0005	0.0152	0.0059	0.0152	0.0097	0.0152	0.0059
	2	0.0099	0.0099	0.0262	0.0262	0.0064	0.0064	0.4233	0.4233	0.5760	0.5760	0.3608	0.3608
	3	0.0050	0.0050	0.0135	0.0135	0.0053	0.0053	0.2028	0.2028	0.1989	0.1989	0.2406	0.2406
	4	0.0033	0.0033	0.0096	0.0096	0.0033	0.0033	0.1636	0.1636	0.1047	0.1047	0.1792	0.1792
	5	0.0062	0.0062	0.0082	0.0082	0.0059	0.0059	0.0969	0.0969	0.0532	0.0532	0.1020	0.1020
0.3	1	0.0576	0.0016	0.0576	0.0054	0.0576	0.0016	0.0576	0.0187	0.0576	0.0337	0.0576	0.0187
	2	0.0084	0.0084	0.0238	0.0238	0.0053	0.0053	0.3977	0.3977	0.5445	0.5445	0.3381	0.3381
	3	0.0049	0.0049	0.0126	0.0126	0.0051	0.0051	0.1954	0.1954	0.1928	0.1928	0.2313	0.2313
	4	0.0031	0.0031	0.0094	0.0094	0.0031	0.0031	0.1594	0.1594	0.1021	0.1021	0.1743	0.1743
	5	0.0060	0.0060	0.0081	0.0081	0.0057	0.0057	0.0942	0.0942	0.0521	0.0521	0.0991	0.0991
0.5	1	0.1498	0.0027	0.1498	0.0113	0.1498	0.0027	0.1498	0.0417	0.1498	0.0801	0.1498	0.0417
	2	0.0064	0.0064	0.0187	0.0187	0.0038	0.0038	0.3470	0.3470	0.4800	0.4800	0.2934	0.2934
	3	0.0039	0.0039	0.0108	0.0108	0.0040	0.0040	0.1807	0.1807	0.1782	0.1782	0.2129	0.2129
	4	0.0026	0.0026	0.0083	0.0083	0.0025	0.0025	0.1455	0.1455	0.0951	0.0951	0.1588	0.1588
	5	0.0055	0.0055	0.0069	0.0069	0.0053	0.0053	0.0877	0.0877	0.0489	0.0489	0.0923	0.0923

Table 6: Simulation results for $\tilde{k} = 3$ with $\rho = 0.3$

		$\theta^{(B)} = 0$						$\theta^{(B)} = 0.5$					
		Corrected test		Naive test		Group-sequential test		Corrected test		Naive test		Group-sequential test	
$\theta^{(A)}$	Look	Pr(stop)	Pr(reject)	Pr(stop)	Pr(reject)	Pr(stop)	Pr(reject)	Pr(stop)	Pr(reject)	Pr(stop)	Pr(reject)	Pr(stop)	Pr(reject)
-0.3	1	0.0005	0.0000	0.0005	0.0000	0.0005	0.0000	0.0005	0.0002	0.0005	0.0003	0.0005	0.0002
	2	0.0001	0.0000	0.0001	0.0000	0.0001	0.0000	0.0001	0.0001	0.0001	0.0001	0.0001	0.0001
	3	0.0155	0.0155	0.0265	0.0265	0.0083	0.0083	0.6769	0.6769	0.7567	0.7567	0.5864	0.5864
	4	0.0044	0.0044	0.0129	0.0129	0.0040	0.0040	0.1421	0.1421	0.1270	0.1270	0.1986	0.1986
	5	0.0058	0.0058	0.0090	0.0090	0.0061	0.0061	0.0858	0.0858	0.0596	0.0596	0.1084	0.1084
-0.1	1	0.0026	0.0001	0.0026	0.0004	0.0026	0.0001	0.0026	0.0012	0.0026	0.0018	0.0026	0.0012
	2	0.0016	0.0000	0.0016	0.0001	0.0016	0.0000	0.0016	0.0016	0.0016	0.0016	0.0016	0.0012
	3	0.0154	0.0154	0.0263	0.0263	0.0082	0.0082	0.6736	0.6736	0.7533	0.7533	0.5834	0.5834
	4	0.0042	0.0042	0.0126	0.0126	0.0038	0.0038	0.1423	0.1423	0.1269	0.1269	0.1984	0.1984
	5	0.0058	0.0058	0.0090	0.0090	0.0061	0.0061	0.0857	0.0857	0.0596	0.0596	0.1083	0.1083
0	1	0.0063	0.0002	0.0063	0.0007	0.0063	0.0002	0.0063	0.0023	0.0063	0.0041	0.0063	0.0023
	2	0.0053	0.0001	0.0053	0.0003	0.0053	0.0000	0.0053	0.0042	0.0053	0.0044	0.0053	0.0038
	3	0.0152	0.0152	0.0260	0.026	0.0082	0.0082	0.6675	0.6675	0.7469	0.7469	0.5779	0.5779
	4	0.0042	0.0042	0.0125	0.0125	0.0037	0.0037	0.1414	0.1414	0.1262	0.1262	0.1972	0.1972
	5	0.0058	0.0058	0.0090	0.0090	0.0061	0.0061	0.0857	0.0857	0.0595	0.0595	0.1081	0.1081
0.1	1	0.0152	0.0005	0.0152	0.0019	0.0152	0.0005	0.0152	0.0059	0.0152	0.0097	0.0152	0.0059
	2	0.0167	0.0014	0.0167	0.0016	0.0167	0.0007	0.0167	0.0139	0.0167	0.0142	0.0167	0.0119
	3	0.0141	0.0141	0.0246	0.0246	0.0072	0.0072	0.6502	0.6502	0.7287	0.7287	0.5618	0.5618
	4	0.0041	0.0041	0.0119	0.0119	0.0037	0.0037	0.1404	0.1404	0.1253	0.1253	0.1952	0.1952
	5	0.0057	0.0057	0.0086	0.0086	0.0060	0.0060	0.0845	0.0845	0.0586	0.0586	0.1068	0.1068
0.3	1	0.0576	0.0016	0.0576	0.0054	0.0576	0.0016	0.0576	0.0187	0.0576	0.0337	0.0576	0.0187
	2	0.0908	0.0036	0.0908	0.0051	0.0908	0.0012	0.0908	0.0629	0.0908	0.0674	0.0908	0.0486
	3	0.0106	0.0106	0.0193	0.0193	0.0057	0.0057	0.5590	0.5590	0.6300	0.6300	0.4800	0.4800
	4	0.0038	0.0038	0.0105	0.0105	0.0031	0.0031	0.1278	0.1278	0.1156	0.1156	0.1761	0.1761
	5	0.0048	0.0048	0.0071	0.0071	0.0053	0.0053	0.0781	0.0781	0.0543	0.0543	0.0984	0.0984
0.5	1	0.1498	0.0027	0.1498	0.0113	0.1498	0.0027	0.1498	0.0417	0.1498	0.0801	0.1498	0.0417
	2	0.2511	0.0072	0.2511	0.0108	0.2511	0.0020	0.2511	0.1571	0.2511	0.1715	0.2511	0.1172
	3	0.0053	0.0053	0.0099	0.0099	0.0029	0.0029	0.3736	0.3736	0.4240	0.4240	0.3174	0.3174
	4	0.0026	0.0026	0.0061	0.0061	0.0021	0.0021	0.0954	0.0954	0.0899	0.0899	0.1286	0.1286
	5	0.0025	0.0025	0.0044	0.0044	0.0030	0.0030	0.0614	0.0614	0.0431	0.0431	0.0769	0.0769

Table 7: Simulation results for $\tilde{k} = 4$ with $\rho = 0.3$

		$\theta^{(B)} = 0$						$\theta^{(B)} = 0.5$					
		Corrected test		Naive test		Group-sequential test		Corrected test		Naive test		Group-sequential test	
$\theta^{(A)}$	Look	Pr(stop)	Pr(reject)	Pr(stop)	Pr(reject)	Pr(stop)	Pr(reject)	Pr(stop)	Pr(reject)	Pr(stop)	Pr(reject)	Pr(stop)	Pr(reject)
-0.3	1	0.0005	0.0000	0.0005	0.0000	0.0005	0.0000	0.0005	0.0002	0.0005	0.0003	0.0005	0.0002
	2	0.0001	0.0000	0.0001	0.0000	0.0001	0.0000	0.0001	0.0001	0.0001	0.0001	0.0001	0.0001
	3	0.0001	0.0000	0.0001	0.0000	0.0001	0.0000	0.0001	0.0001	0.0001	0.0001	0.0001	0.0001
	4	0.0181	0.0181	0.0272	0.0272	0.0082	0.0082	0.8356	0.8356	0.8661	0.8661	0.7683	0.7683
	5	0.0071	0.0071	0.0104	0.0104	0.0063	0.0063	0.0804	0.0804	0.0728	0.0728	0.1222	0.1222
-0.1	1	0.0026	0.0001	0.0026	0.0004	0.0026	0.0001	0.0026	0.0012	0.0026	0.0018	0.0026	0.0012
	2	0.0016	0.0000	0.0016	0.0001	0.0016	0.0000	0.0016	0.0016	0.0016	0.0016	0.0016	0.0012
	3	0.0011	0.0001	0.0011	0.0001	0.0011	0.0000	0.0011	0.0011	0.0011	0.0011	0.0011	0.0009
	4	0.0177	0.0177	0.0267	0.0267	0.0078	0.0078	0.8315	0.8315	0.8617	0.8617	0.7642	0.7642
	5	0.0071	0.0071	0.0103	0.0103	0.0063	0.0063	0.0803	0.0803	0.0727	0.0727	0.1221	0.1221
0	1	0.0063	0.0002	0.0063	0.0007	0.0063	0.0002	0.0063	0.0023	0.0063	0.0041	0.0063	0.0023
	2	0.0053	0.0001	0.0053	0.0003	0.0053	0.0000	0.0053	0.0042	0.0053	0.0044	0.0053	0.0038
	3	0.0048	0.0005	0.0048	0.0005	0.0048	0.0001	0.0048	0.0046	0.0048	0.0046	0.0048	0.0041
	4	0.0176	0.0176	0.0264	0.0264	0.0077	0.0077	0.8210	0.8210	0.8510	0.8510	0.7541	0.7541
	5	0.0070	0.0070	0.0101	0.0101	0.0063	0.0063	0.0801	0.0801	0.0725	0.0725	0.1216	0.1216
0.1	1	0.0152	0.0005	0.0152	0.0019	0.0152	0.0005	0.0152	0.0059	0.0152	0.0097	0.0152	0.0059
	2	0.0167	0.0014	0.0167	0.0016	0.0167	0.0007	0.0167	0.0139	0.0167	0.0142	0.0167	0.0119
	3	0.0191	0.0016	0.0191	0.0016	0.0191	0.0005	0.0191	0.0178	0.0191	0.0178	0.0191	0.0147
	4	0.0155	0.0155	0.0233	0.0233	0.0067	0.0067	0.7893	0.7893	0.8187	0.8187	0.7236	0.7236
	5	0.0064	0.0064	0.0094	0.0094	0.0059	0.0059	0.0781	0.0781	0.0708	0.0708	0.1191	0.1191
0.3	1	0.0576	0.0016	0.0576	0.0054	0.0576	0.0016	0.0576	0.0187	0.0576	0.0337	0.0576	0.0187
	2	0.0908	0.0036	0.0908	0.0051	0.0908	0.0012	0.0908	0.0629	0.0908	0.0674	0.0908	0.0486
	3	0.1062	0.0065	0.1062	0.0064	0.1062	0.0016	0.1062	0.0919	0.1062	0.0918	0.1062	0.0782
	4	0.0099	0.0099	0.0144	0.0144	0.0040	0.0040	0.6061	0.6061	0.6309	0.6309	0.5502	0.5502
	5	0.0045	0.0045	0.0059	0.0059	0.0041	0.0041	0.0673	0.0673	0.0617	0.0617	0.1018	0.1018
0.5	1	0.1498	0.0027	0.1498	0.0113	0.1498	0.0027	0.1498	0.0417	0.1498	0.0801	0.1498	0.0417
	2	0.2511	0.0072	0.2511	0.0108	0.2511	0.0020	0.2511	0.1571	0.2511	0.1715	0.2511	0.1172
	3	0.2321	0.0065	0.2321	0.0062	0.2321	0.0019	0.2321	0.1829	0.2321	0.1819	0.2321	0.1434
	4	0.0027	0.0027	0.0042	0.0042	0.0009	0.0009	0.2807	0.2807	0.2948	0.2948	0.2495	0.2495
	5	0.0018	0.0018	0.0024	0.0024	0.0016	0.0016	0.0421	0.0421	0.0380	0.0380	0.0610	0.0610

Table 8: Simulation results for $\tilde{k} = 5$ with $\rho = 0.3$

		$\theta^{(B)} = 0$						$\theta^{(B)} = 0.5$					
		Corrected test		Naive test		Group-sequential test		Corrected test		Naive test		Group-sequential test	
$\theta^{(A)}$	Look	Pr(stop)	Pr(reject)	Pr(stop)	Pr(reject)	Pr(stop)	Pr(reject)	Pr(stop)	Pr(reject)	Pr(stop)	Pr(reject)	Pr(stop)	Pr(reject)
-0.3	1	0.0005	0.0000	0.0005	0.0000	0.0005	0.0000	0.0005	0.0002	0.0005	0.0003	0.0005	0.0002
	2	0.0001	0.0000	0.0001	0.0000	0.0001	0.0000	0.0001	0.0001	0.0001	0.0001	0.0001	0.0001
	3	0.0001	0.0000	0.0001	0.0000	0.0001	0.0000	0.0001	0.0001	0.0001	0.0001	0.0001	0.0001
	4	0.0001	0.0000	0.0001	0.0000	0.0001	0.0000	0.0001	0.0001	0.0001	0.0001	0.0001	0.0001
	5	0.0231	0.0231	0.0262	0.0262	0.0109	0.0109	0.9223	0.9223	0.9298	0.9298	0.8817	0.8817
-0.1	1	0.0026	0.0001	0.0026	0.0004	0.0026	0.0001	0.0026	0.0012	0.0026	0.0018	0.0026	0.0012
	2	0.0016	0.0000	0.0016	0.0001	0.0016	0.0000	0.0016	0.0016	0.0016	0.0016	0.0016	0.0012
	3	0.0011	0.0001	0.0011	0.0001	0.0011	0.0000	0.0011	0.0011	0.0011	0.0011	0.0011	0.0009
	4	0.0008	0.0002	0.0008	0.0001	0.0008	0.0000	0.0008	0.0008	0.0008	0.0008	0.0008	0.0008
	5	0.0227	0.0227	0.0258	0.0258	0.0108	0.0108	0.9175	0.9175	0.9247	0.9247	0.8768	0.8768
0	1	0.0063	0.0002	0.0063	0.0007	0.0063	0.0002	0.0063	0.0023	0.0063	0.0041	0.0063	0.0023
	2	0.0053	0.0001	0.0053	0.0003	0.0053	0.0000	0.0053	0.0042	0.0053	0.0044	0.0053	0.0038
	3	0.0048	0.0005	0.0048	0.0005	0.0048	0.0001	0.0048	0.0046	0.0048	0.0046	0.0048	0.0041
	4	0.0062	0.0012	0.0062	0.0007	0.0062	0.0003	0.0062	0.0062	0.0062	0.0061	0.0062	0.0059
	5	0.0220	0.0220	0.0248	0.0248	0.0107	0.0107	0.9014	0.9014	0.9085	0.9085	0.8610	0.8610
0.1	1	0.0152	0.0005	0.0152	0.0019	0.0152	0.0005	0.0152	0.0059	0.0152	0.0097	0.0152	0.0059
	2	0.0167	0.0014	0.0167	0.0016	0.0167	0.0007	0.0167	0.0139	0.0167	0.0142	0.0167	0.0119
	3	0.0191	0.0016	0.0191	0.0016	0.0191	0.0005	0.0191	0.0178	0.0191	0.0178	0.0191	0.0147
	4	0.0206	0.0021	0.0206	0.0017	0.0206	0.0009	0.0206	0.0200	0.0206	0.0195	0.0206	0.0190
	5	0.0194	0.0194	0.0218	0.0218	0.0091	0.0091	0.8533	0.8533	0.8604	0.8604	0.8141	0.8141
0.3	1	0.0576	0.0016	0.0576	0.0054	0.0576	0.0016	0.0576	0.0187	0.0576	0.0337	0.0576	0.0187
	2	0.0908	0.0036	0.0908	0.0051	0.0908	0.0012	0.0908	0.0629	0.0908	0.0674	0.0908	0.0486
	3	0.1062	0.0065	0.1062	0.0064	0.1062	0.0016	0.1062	0.0919	0.1062	0.0918	0.1062	0.0782
	4	0.1189	0.0064	0.1189	0.0048	0.1189	0.0016	0.1189	0.1117	0.1189	0.1095	0.1189	0.1011
	5	0.0092	0.0092	0.0102	0.0102	0.0044	0.0044	0.5655	0.5655	0.5707	0.5707	0.5369	0.5369
0.5	1	0.1498	0.0027	0.1498	0.0113	0.1498	0.0027	0.1498	0.0417	0.1498	0.0801	0.1498	0.0417
	2	0.2511	0.0072	0.2511	0.0108	0.2511	0.0020	0.2511	0.1571	0.2511	0.1715	0.2511	0.1172
	3	0.2321	0.0065	0.2321	0.0062	0.2321	0.0019	0.2321	0.1829	0.2321	0.1819	0.2321	0.1434
	4	0.1608	0.0043	0.1608	0.0028	0.1608	0.0006	0.1608	0.1446	0.1608	0.1389	0.1608	0.1206
	5	0.0017	0.0017	0.0018	0.0018	0.0007	0.0007	0.1785	0.1785	0.1806	0.1806	0.1657	0.1657
